# EGFR Regulates the Hippo pathway by promoting the tyrosine phosphorylation of MOB1

**DOI:** 10.1038/s42003-021-02744-4

**Published:** 2021-11-01

**Authors:** Toshinori Ando, Nadia Arang, Zhiyong Wang, Daniela Elena Costea, Xiaodong Feng, Yusuke Goto, Hiroki Izumi, Mara Gilardi, Kazuyo Ando, J. Silvio Gutkind

**Affiliations:** 1 Moores Cancer Center, University of California, San Diego, La Jolla, CA USA; 2 Graduate School of Biomedical and Health Sciences, Hiroshima University, Hiroshima, Japan; 3 Department of Pharmacology, University of California, San Diego, La Jolla, CA USA; 4 Department of Clinical Medicine and Centre for Cancer Biomarkers CCBio, Faculty of Medicine, University of Bergen, Bergen, Norway; 5 Department of Pathology, Haukeland University Hospital, Bergen, Norway; 6 Department of Orthodontics, Applied Life Sciences, Hiroshima University Institute of Biomedical & Health Sciences, Hiroshima, Japan

**Keywords:** Growth factor signalling, Targeted therapies

## Abstract

The Hippo pathway is frequently dysregulated in cancer, leading to the unrestrained activity of its downstream targets, YAP/TAZ, and aberrant tumor growth. However, the precise mechanisms leading to YAP/TAZ activation in most cancers is still poorly understood. Analysis of large tissue collections revealed YAP activation in most head and neck squamous cell carcinoma (HNSCC), but only 29.8% of HNSCC cases present genetic alterations in the *FAT1* tumor suppressor gene that may underlie persistent YAP signaling. EGFR is overexpressed in HNSCC and many other cancers, but whether EGFR controls YAP activation is still poorly understood. Here, we discover that EGFR activates YAP/TAZ in HNSCC cells, but independently of its typical signaling targets, including PI3K. Mechanistically, we find that EGFR promotes the phosphorylation of MOB1, a core Hippo pathway component, and the inactivation of LATS1/2 independently of MST1/2. Transcriptomic analysis reveals that erlotinib, a clinical EGFR inhibitor, inactivates YAP/TAZ. Remarkably, loss of LATS1/2, resulting in aberrant YAP/TAZ activity, confers erlotinib resistance on HNSCC and lung cancer cells. Our findings suggest that EGFR-YAP/TAZ signaling plays a growth-promoting role in cancers harboring EGFR alterations, and that inhibition of YAP/TAZ in combination with EGFR might be beneficial to prevent treatment resistance and cancer recurrence.

## Abstract (web summary)

Ando et al show in head and neck squamous cell carcinoma cells that EGFR activation leads to the phosphorylation of the Hippo pathway component, MOB1 to inhibit LATS1/2 function resulting in YAP/TAZ activation. Further, EGFR-targeting therapies suppress YAP/TAZ, and loss of LATS1/2-mediated YAP/TAZ activation confers therapy resistance, thus offering insights into potential drug resistance mechanisms in cancers with activated EGFR.

## Introduction

The Hippo pathway is a tumor-suppressive signaling route and its downstream targets, Yes-associated protein (YAP) and transcriptional co-activator with PDZ binding motif (TAZ), play a central role in normal tissue growth and organ size^1^. In mammals, the core Hippo kinase pathway consists of mammalian STE20-like kinase 1 and 2 (MST1/2), large tumor suppressor 1 and 2 (LATS1/2), and their adaptor proteins salvador homologue 1 (SAV1) and MOB kinase activator 1A and 1B (MOB1A/B, hereafter MOB1), respectively^2^. MST1/2 phosphorylate the hydrophobic motif of LATS1/2, and subsequently activated LATS1/2 phosphorylate YAP on multiple serine residues (pYAP), leading to its cytoplasmic retention by binding to 14-3-3 and/or degradation through the ubiquitin-proteasome pathway^3^. In the absence of Hippo pathway signaling, LATS1/2 inactivation results in nuclear translocation of hypo-phosphorylated YAP and its interaction with transcription factors including TEA domain family members (TEAD) to enhance the transcription of growth-related genes^4^. YAP/TAZ are aberrantly activated in many types of cancer^5^, including head and neck squamous cell carcinomas (HNSCC), a disease that is diagnosed in around 65,410 new cases each year in the United States alone, resulting in more than 14,620 deaths^6^.

The mechanisms resulting in YAP/TAZ activation in most cancer types are still poorly understood. Specifically for HNSCC, The Cancer Genome Atlas (TCGA) has provided a comprehensive landscape of somatic genomic alterations in this cancer type^7^, which revealed that HNSCC is among the cancers showing the highest incidence of *YAP1* gene amplification (6.3% of the cases). In addition, our recent study has uncovered that HNSCCs have frequent alterations of *FAT1* (29.8%), which results in YAP activation and its consequent YAP-dependent tumor growth^8^. FAT1 assembles a multimeric Hippo pathway signaling complex, inducing activation of core Hippo kinases by TAO kinases resulting in YAP inactivation^8^. However, it is still possible that other molecular events may control YAP activation in >65% of HNSCC cases that do not exhibit *YAP1* or *FAT1* genomic alterations, whose elucidation may help reveal new molecular mechanisms controlling the Hippo pathway in cancer.

In this regard, EGFR, one of the ERBB family tyrosine kinases, is amplified and highly overexpressed in HNSCC and lung squamous cell carcinoma, and mutated and activated in many cancer types including lung adenocarcinoma and glioblastoma^7,9–11^. Therefore, EGFR is a widely accepted therapeutic target, either using small molecule tyrosine kinase inhibitors (e.g., erlotinib in lung adenocarcinoma) or blocking antibody (e.g., cetuximab in HNSCC). The link between EGFR activation and the Hippo pathway is still poorly understood^12^, with EGFR failing to reduce the phosphorylation of YAP at S127 and nuclear localization in some cellular systems^13,14^, but inhibiting the Hippo pathway to activate YAP in others^15–18^.

Here, we show that EGFR activation leads to the phosphorylation of one of the core Hippo pathway components, MOB1 to inhibit LATS1/2 function thus resulting in YAP/TAZ activation in HNSCC cells independent of *FAT1* alterations. Remarkably, EGFR-targeting therapies suppress YAP/TAZ, and loss of LATS1/2-mediated YAP/TAZ activation confers therapy resistance. These findings contribute to the understanding of the mechanisms by which EGFR-driven signaling networks control YAP/TAZ activation in normal cells and cancer, and support the therapeutic potential of inhibiting YAP/TAZ function in patients with cancers harboring EGFR alterations to enhance the response to EGFR targeted therapies, and prevent emergence of drug resistance.

## Results

### EGFR activates YAP/TAZ in HNSCC cells, independently of PI3K

**Fig. 1:**
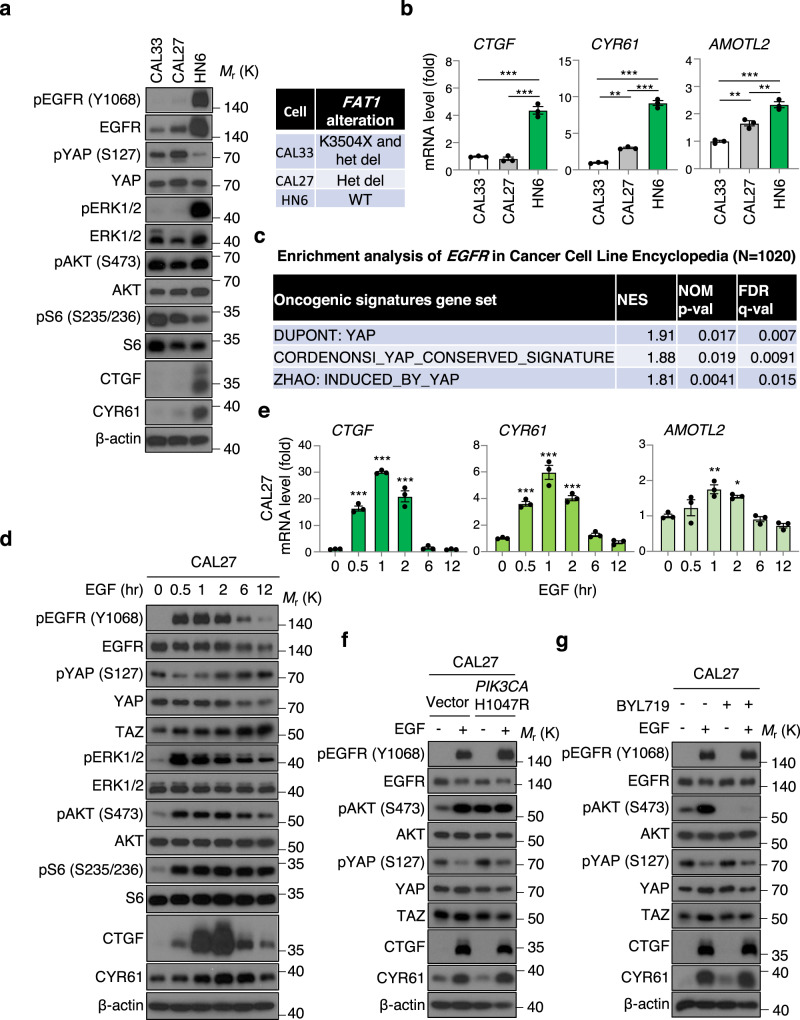
EGFR activates YAP/TAZ in HNSCC, independently of PI3K. **a** Immunoblot of pEGFR (Y1068), EGFR, pYAP (S127), YAP, pERK1/2 (T202/Y204), ERK1/2, pAKT (S473), AKT, pS6 (S235/236), S6, CTGF, CYR61, β-actin in CAL33, CAL27, and WSU-HN6. Right panel showing the status of *FAT1* gene alterations. **b** Relative mRNA levels of *CTGF*, *CYR61*, and *AMOTL2* in CAL33, CAL27, and WSU-HN6 cells. **c** GSEA analysis of RNA-seq data in CCLE using the C6 oncogenic gene sets, spiked with several previously published YAP-regulated gene sets. NES, normalized enrichment score; NOM, nominal; FDR, false discovery rate. **d** Immunoblot of pEGFR (Y1068), EGFR, pYAP (S127), YAP, TAZ, pERK1/2 (T202/Y204), ERK1/2, pAKT (S473), AKT, pS6 (S235/236), S6, CTGF, CYR61, β-actin in CAL27 cells. Cells were serum starved for 16 h, and treated with EGF (20 ng/ml) for the indicated time. **e** Relative mRNA levels of *CTGF*, *CYR61*, and *AMOTL2* in CAL27 cells. **f** Immunoblot of pEGFR (Y1068), EGFR, pAKT (S473), AKT, pYAP (S127), YAP, TAZ, CTGF, CYR61, β-actin in CAL27 cells stably overexpressing empty vector or *PIK3CA* H1047R. Cells were serum starved for 16 h, and treated with EGF (20 ng/ml) for 1 hr. **g** Immunoblot of pEGFR (Y1068), EGFR, pAKT (S473), AKT, pYAP (S127), YAP, TAZ, CTGF, CYR61, β-actin in CAL27 cells. Cells were serum starved for 16 h, and pretreated with BYL719 (1 μM) for 1 h and followed by EGF treatment (20 ng/ml) for 1 h. ANOVA with Tukey–Kramer post hoc test was used. Mean ± SEM (**b**, **e**); ***P  < 0.001; **P < 0.01; *P  < 0.05. *versus CAL33 (**b**) and versus EGF 0 h (**e**).

We have recently reported that frequent *FAT1* alterations contribute to YAP activation in HNSCC, however many *FAT1* wild type HNSCC cases also exhibit nuclear YAP^8^, and as such, the mechanism of YAP activation in HNSCC, and other cancer types, may not yet be fully understood. As a potential upstream activating component, we focused on EGFR, because it is overexpressed or amplified in most HNSCC cases^7^, and the target of the only approved cancer-targeting therapy in this malignancy^19,20^. We first compared EGFR expression and YAP activation among HNSCC cell lines including CAL33 that harbors hemizygous *FAT1* K3504X mutation and loss of the remaining allele, and CAL27 cells that have one remaining *FAT1* copy^8^. We also used WSU-HN6 cells (herein referred as HN6), which show the highest EGFR expression among our HNSCC cell line panel, but lack *FAT1* alterations^21^. Remarkably, HN6 cells showed lower pYAP level and higher expression of YAP-regulated genes *CTGF, CYR61*, and *AMOTL2*, and the CTGF and CYR61 protein products (Fig. 1a and b). We extended this analysis to all cancer types using the Cancer Cell Line Encyclopedia (CCLE) data set (1020 cancer cell lines^22^). Gene set enrichment analysis (GSEA) revealed that YAP-regulated signatures gene sets (DUPONT: YAP, CORDENONSI_YAP_CONSERVED_SIGNATURE, ZHAO: INDUCED_BY_YAP) including representative YAP-regulated genes (e.g., *CTGF, CYR61, AMOTL2*) were enriched with higher *EGFR* expression (Fig. 1c, Supplementary Figs. S1, 2a and b). In addition, when HNSCC patients from TCGA were stratified based on mRNA expression of *EGFR*, *CTGF*, and *CYR61* (all *z*-score > 0 vs all *z*-score < 0), the *EGFR*, *CTGF*, and *CYR61* “high” group (co-overexpression of EGFR and representative YAP-target genes) showed poorer survival with respect to those patients expressing low levels (high group: *n* = 128, low group: *n* = 114, Log-rank *P* = 0.015, Genhan-Breslow–Wilcoxon *P* = 0.0075). (Supplementary Fig. S3a). This suggests that EGFR-activated YAP/TAZ correlates with poor patient survival.

We next looked to examine whether EGFR can activate YAP/TAZ, CAL27 cells were treated with EGF. EGF treatment reduced pYAP and increased TAZ levels, as well as *CTGF, CYR61, AMOTL2* mRNA and CTGF and CYR61 protein expression, concomitant with the activation of canonical EGFR-downstream pathways including MAPK and PI3K-AKT-mTOR, reflected by increased phosphorylation of ERK1/2, AKT, and S6 (Fig. 1d and e). Similar results were observed in CAL33 cells, which showed pYAP reduction and increase in YAP/TAZ transcriptional targets (Supplementary Fig. S3b and c). Therefore, EGFR can further activate YAP/TAZ even in the cells harboring *FAT1* alterations.

Several reports have shown that PDK1 can form complex with the Hippo components including MST1/2, SAV1, LATS1/2, and PDK1 recruited to the plasma membrane triggered by PI3K leads to dissociation of these complex and YAP activation in MCF-10A, HEK293T, and hepatocellular carcinoma cells^15,16^. Activated PI3K phosphorylates phosphatidylinositol (3,4)-bisphosphate (PIP_2_), inducing conversion of PIP_2_ to phosphatidylinositol (3,4,5)-triphosphate (PIP_3_). PIP_3_ recruits PDK1 and AKT to the plasma membrane, enabling PDK1 to access and phosphorylate AKT^23^. To investigate whether PDK1 mediates EGFR-YAP signaling, we overexpressed constitutive active *PIK3CA* (H1047R) to promote PDK1 activation, which was reflected by AKT phosphorylation (Fig. 1f). However, constitutive active *PIK3CA* (H1047R) overexpression did not show reduced pYAP and expression of YAP/TAZ regulated molecules, nor potentiated EGF-induced effects on this pathway and CTGF/CYR61 expression in HNSCC cells (Fig. 1f). Moreover, BYL719, a PI3Kα inhibitor, abolished AKT phosphorylation, but failed to suppress EGFR-induced pYAP reduction and CTGF/CYR61 production (Fig. 1g). Collectively, our findings suggest that EGFR can activate YAP/TAZ independently of *FAT1* alterations and PI3K signaling in HNSCC cells.

### Reconstituted EGFR expression induces hypo-phosphorylation and nuclear translocation of YAP/TAZ, thereby enhancing transcription of YAP/TAZ-regulated genes

**Fig. 2:**
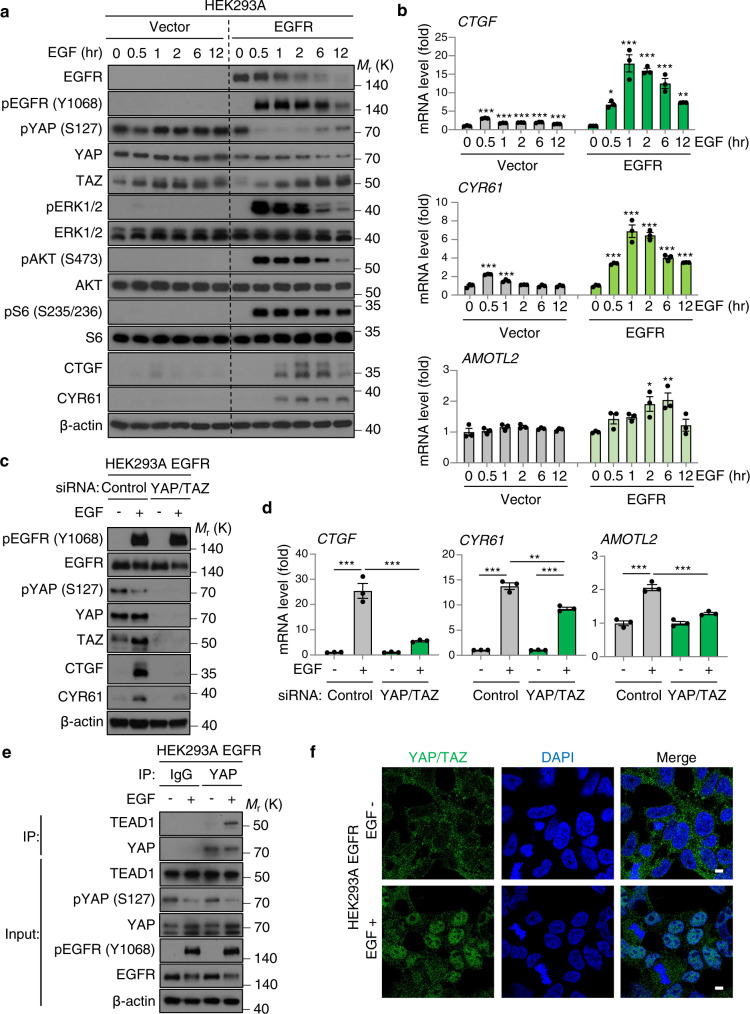
EGFR under-phosphorylates YAP/TAZ, induces nuclear translocation of YAP/TAZ and their interaction with TEADs, promoting *CTGF*/*CYR61*/*AMOTL2* expression. **a** Immunoblot of EGFR, pEGFR (Y1068), pYAP (S127), YAP, TAZ, pERK1/2 (T202/Y204), ERK1/2, pAKT (S473), AKT, pS6 (S235/236), S6, CTGF, CYR61, β-actin in vector- or EGFR-overexpressing HEK293A cells. Cells were serum starved for 16 h, and treated with EGF (20 ng/ml) for the indicated time. **b** Relative mRNA levels of *CTGF*, *CYR61*, and *AMOTL2*. **c** Immunoblot of pEGFR (Y1068), EGFR, pYAP (S127), YAP, TAZ, CTGF, CYR61, β-actin in EGFR-overexpressing HEK293A cells. Cells were transfected with siRNA control and against YAP/TAZ for 24 h, serum starved for 16 h, and treated with EGF (20 ng/ml) for 1 h. **d** Relative mRNA levels of *CTGF*, *CYR61*, and *AMOTL2*. **e** Co-immunoprecipitation of YAP and TEAD1. Lysates were immunoprecipitated with control IgG or an antibody against YAP. Immunoblot of TEAD1, YAP, pYAP (S127), pEGFR (Y1068), EGFR, β-actin in EGFR-overexpressing HEK293A cells. Cells were serum stared for 16 h, and treated with EGF (20 ng/ml) for 1 h. **f** YAP/TAZ localization analyzed by immunofluorescence staining. Cells were serum starved for 16 h, and treated with EGF (20 ng/ml) for 1 h. Scale bars indicate 5 μm. ANOVA with Tukey–Kramer post hoc test was used. Mean ± SEM (**b**, **d**); ***P <0.001; **P <0.01; *P <0.05. *versus EGF 0 h (**b**).

To examine the precise mechanism by which EGFR activates YAP/TAZ, we established EGFR-overexpressing HEK293A cells, recapitulating HNSCC and other EGFR overexpressing cancer types. Vector-expressing HEK293A cells showed almost no effect on YAP by EGF treatment, but EGFR-overexpressing HEK293A showed significant pYAP reduction concomitant with *CTGF*, *CYR61*, and *AMOTL2* mRNA increase, as well as ERK1/2, AKT, and S6 phosphorylation (Fig. 2a and b). *PIK3CA* WT and H1047R overexpression slightly increased YAP/TAZ and CTGF/CYR61 more than those of vector alone, however, BYL719 treatment failed to inhibit EGFR-induced pYAP reduction, TAZ increase, and CTGF and CYR61 protein expression (Supplementary Fig. S4a and b), which is consistent with our prior results in HNSCC cells (Fig. 1f and g). Moreover, knockdown of YAP/TAZ significantly inhibited EGFR-induced *CTGF*, *CYR61*, and *AMOTL2* expression (Fig. 2c and d).

Under-phosphorylated and activated YAP/TAZ translocate from cytoplasm into nucleus, where they bind to TEAD transcription factor to act as a co-activator enhancing the transcription of proliferation-related genes^4^. EGFR induced hypo-phosphorylation of YAP and increased YAP-TEAD1 interaction (Fig. 2e), and immunofluorescence staining showed that EGFR activation triggered YAP/TAZ translocation from the cytoplasm into nucleus (Fig. 2f and supplementary Fig. S4c). In summary, in EGFR expressing cells EGF activation induces hypo-phosphorylation of YAP and stabilization of TAZ, promote nuclear translocation of YAP/TAZ and their interaction with TEADs, which results in increased transcription of their target genes *CTGF*, *CYR61*, and *AMOTL2*.

### EGFR promotes the phosphorylation of MOB1 and LATS1/2 inactivation, independently of MST1/2

**Fig. 3:**
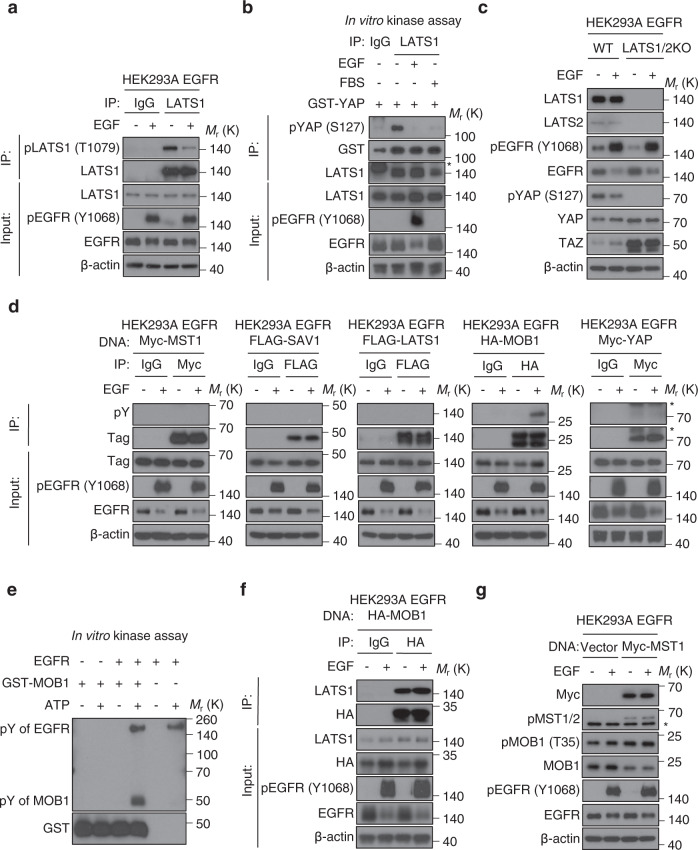
EGFR stimulation leads to MOB1 phosphorylation and LATS1/2 inactivation, independently of MST1/2. **a** Immunoprecipitation of LATS1. Lysates were immunoprecipitated with control IgG or an antibody against LATS1. Immunoblot of pLATS1 (T1079), LATS1, pEGFR (Y1068), EGFR, β-actin in EGFR-overexpressing HEK293A cells. Cells were serum stared for 16 h, and treated with EGF (20 ng/ml) for 1 h. **b** In vitro kinase assay of LATS1 against YAP. Lysates were immunoprecipitated with control IgG or an antibody against LATS1, then applied for in vitro kinase reaction with GST-YAP protein. Cells were serum starved for 16 h, and treated with EGF (20 ng/ml) or FBS (10%) as positive control for 1 h. Immunoblot of pYAP (S127), GST, LATS1, pEGFR (Y1068), EGFR, β-actin. Arrow indicates non-specific band. **c** Immunoblot of LATS1, LATS2, pEGFR (Y1068), EGFR, pYAP (S127), YAP, TAZ, β-actin in WT, or LATS1/2 KO HEK293A cells. WT or LATS1/2 KO HEK293A cells were transfected with EGFR plasmid and incubated for 24 h, serum starved for 16 h, and treated with EGF (20 ng/ml) for 1 h. **d** Immunoprecipitation of Myc-MST1, FLAG-SAV1, FLAG-LATS1, HA-MOB1, and Myc-YAP. Lysates were immunoprecipitated with control IgG or antibodies against each Tag. Immunoblot of pY, Tag, pEGFR (Y1068), EGFR, β-actin. EGFR-overexpressing HEK293A cells were transfected with the Hippo-components and YAP plasmid, incubated for 24 h, serum starved for 16 h, and treated with EGF (20 ng/ml) for 1 h. **e** In vitro kinase assay of EGFR and GST-MOB1. In vitro kinase reaction was performed with recombinant EGFR, GST-MOB1 protein, and ATP. Immunoblot for pY and GST. **f** Co-immunoprecipitation of HA-MOB1 and LATS1. Lysates were immunoprecipitated with control IgG or an antibody against HA-tag. Immunoblot of LATS1, HA, pEGFR (Y1068), EGFR, β-actin. EGFR-overexpressing HEK293A cells were transfected with HA-MOB1 plasmid and incubated for 24 h, serum starved for 16 h, and treated with EGF (20 ng/ml) for 1 h. **g** Immunoblot of Myc-tag, pMST1/2 (T183/T180), pMOB1 (T35), MOB1, pEGFR (Y1068), EGFR, β-actin. EGFR-overexpressing HEK293A cells were transfected with vector or Myc-MST1 plasmid and incubated for 24 h, serum starved for 16 h, and treated with EGF (20 ng/ml) for 1 h. Asterisks indicate non-specific bands.

Next, we sought to understand the mechanism of how EGFR activates YAP/TAZ. Because LATS1/2 directly phosphorylate YAP/TAZ on serine residues leading to cytoplasmic retention or proteosomal degradation, we examined LATS1/2 activity in the context of EGFR activation. The phosphorylation of the hydrophobic motif of LATS1/2 on threonine 1079 (T1079), which reflects its activity^24^, was reduced by EGFR activation, suggesting that LATS1/2 were inactivated (Fig. 3a). Indeed, in vitro kinase assays showed that LATS1 kinase activity on YAP was suppressed by EGF treatment (Fig. 3b). FBS was used as a positive control, as lysophosphatidic acid (LPA) and sphingosine 1-phosphophate (S1P) in serum inactivate LATS1/2 thereby stimulate YAP through G12/13-coupled receptors^13^. In addition, CRISPR/Cas9 engineered LATS1/2 knockout cells abolished pYAP with or without EGF treatment, and increased TAZ level, and the status of YAP/TAZ was not changed further by EGFR activation (Fig. 3c). These data support that LATS1/2 are inactivated by EGFR, thereby promoting YAP activity.

We next attempted to clarify how LATS1/2 activity is suppressed by EGFR activation. Recent studies suggest that Hippo components can be regulated through tyrosine phosphorylation. For example, MST1 can be phosphorylated by FGFR4 and c-Abl, LATS1 by Src, and MOB1 by FAK^25–28^. Thus, we hypothesized that EGFR stimulation may lead to the phosphorylation of Hippo kinase components to activate YAP/TAZ. To test this hypothesis, we examined tyrosine phosphorylation of MST1, SAV1, LATS1, MOB1, and YAP by EGFR activation. Interestingly, only MOB1 showed tyrosine phosphorylation upon EGFR stimulation in cell in vivo (Fig. 3d). In vitro kinase assays showed that EGFR can directly phosphorylate MOB1 (Fig. 3e), but to explore whether this is also the case in cells in vivo we tested whether MOB1 associates with EGFR by co-immunoprecipitation assays. Although GRB2, an adapter protein acting directly downstream of EGFR, associated tightly with EGFR upon EGF stimulation, MOB1 association with EGFR or GRB2 could not be detected (Supplementary Fig. S4d). Thus, MOB1 may represent a downstream substrate of EGFR without forming stable protein complexes, which is aligned with the absence of recognizable EGFR-interaction motifs in MOB1, or alternatively, MOB1 may be phosphorylated downstream of EGFR through intermediated receptor or non-receptor tyrosine kinases. Because MOB1 acts as an adaptor protein for LATS1/2, we examined the status of association of MOB1 with LATS1, which was not disrupted by EGFR activation (Fig. 3f). Moreover, since the hydrophobic motif of LATS1/2 is phosphorylated by MST1/2, we examined the activity of MST1/2. Phosphorylation of MST1/2 on threonine 180 and 183 autophosphorylation sites, reflecting MST1/2 activity, did not show differences upon EGFR activation. In addition, phosphorylation of threonine (T)35 of MOB1, a target site of MST1/2, was not affected by EGFR stimulation (Fig. 3g). These data suggest that EGFR activation promotes MOB1 phosphorylation, thus leading to LATS1/2 inactivation but independently of MST1/2.

### Tyrosine phosphorylation of MOB1 suppresses its function

**Fig. 4:**
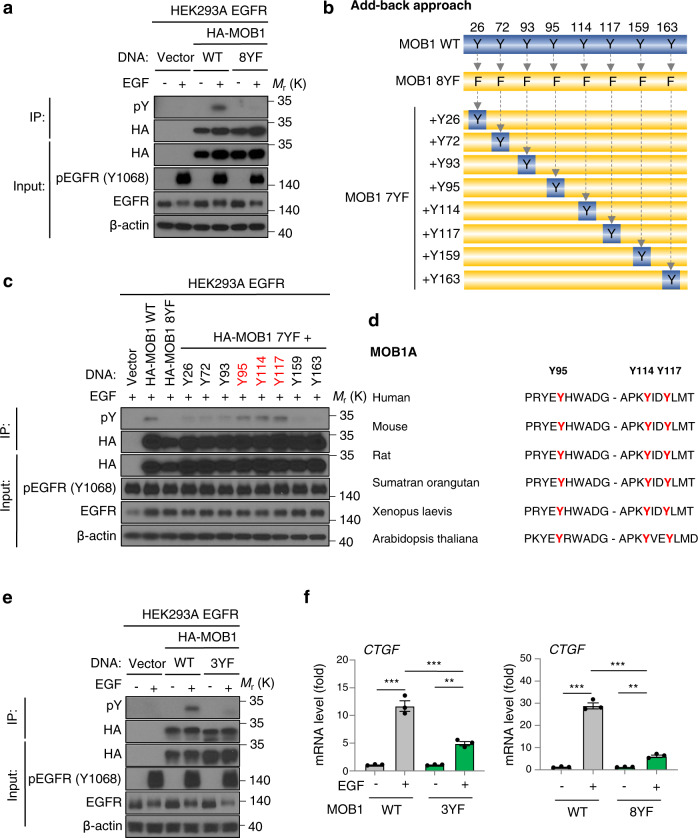
EGFR activation leads to MOB1 phosphorylation at Y95, 114, 117. **a** Immunoprecipitation of HA-MOB1 WT and 8YF. Lysates were immunoprecipitated with an antibody against HA-tag. Immunoblot of pY, HA, pEGFR (Y1068), EGFR, β-actin. EGFR-overexpressing HEK293A cells were transfected with HA-MOB1 WT or 8YF plasmid and incubated for 24 h, serum starved for 16 h, and treated with EGF (20 ng/ml) for 1 h. **b** Schematic of “Add-back approach”. All 8 tyrosines of MOB1 WT were mutated into phenylalanine (8YF), then each site was mutated back to tyrosine (7YF + Y). **c** Immunoprecipitation of HA-MOB1 WT, 8YF and 7YF + Y26, Y72, Y93, Y95, Y114, Y117, Y159, Y163. Lysates were immunoprecipitated with an antibody against HA-tag. Immunoblot of pY, HA, pEGFR (Y1068), EGFR, β-actin. EGFR-overexpressing HEK293A cells were transfected with HA-MOB1 WT, 8YF, and 7YF + Y mutant plasmid and incubated for 24 h, serum starved for 16 h, and treated with EGF (20 ng/ml) for 1 h. **d** The conserved amino acid sequences at Y95, Y114, Y117 of MOB1A in various species. **e** Immunoprecipitation of HA-MOB1 WT and 3YF. Lysates were immunoprecipitated with an antibody against HA-tag. Immunoblot of pY, HA, pEGFR (Y1068), EGFR, β-actin. EGFR-overexpressing HEK293A cells were transfected with HA-MOB1 WT or 3YF plasmid and incubated for 24 h, serum starved for 16 h, and treated with EGF (20 ng/ml) for 1 h. **f** Relative mRNA expression of *CTGF*. ANOVA with Tukey–Kramer post hoc test was used. Mean ± SEM (**f**); ***P <0.001; **P <0.01.

We have recently shown that FAK phosphorylates MOB1 on Y26^28^. However, EGFR activation failed to increase pY26 of MOB1, suggesting the other tyrosine residues might be phosphorylated downstream from EGFR (Supplementary Fig. S4e). In order to determine the MOB1 phosphorylation sites, all 8 tyrosine sites of MOB1 were mutated into an unphosphorylatable amino acid, phenylalanine (MOB1 8YF). As expected, while MOB1 WT was phosphorylated by EGFR activation, MOB1 8YF was not (Fig. 4a). We then performed an “add-back approach”; each site was mutated back to tyrosine individually (MOB1 7YF + Y26, Y72, Y93, Y95, Y114, Y117, Y159, and Y163) (Fig. 4b). Only MOB1 7YF + Y95, Y114, and Y117 showed phosphorylation by EGFR (Fig. 4c). MOB1 Y95, Y114, and Y117 residues are well conserved among species (Fig. 4d). Based on the results, we mutated Y95, Y114, and Y117 of MOB1 into phenylalanine (MOB1 3YF) and confirmed that it failed to be phosphorylated by EGFR (Fig. 4e). Both MOB1 3YF and 8YF suppressed EGFR-enhanced *CTGF* expression in comparison with MOB1 WT, and MOB1 3YF showed reduction of CYR61 as well (Fig. 4f and Supplementary Fig. S4f, g). Additionally, we tested pLATS1 status in MOB1 WT and MOB1 8YF overexpressing cells. EGF treatment reduced pLATS1 (T1079) in cells expressing MOB1 WT, but pLATS1 in MOB1 8YF expressing cells remained higher than those of MOB1 WT (Supplementary Fig. S4h). In short, EGFR phosphorylates MOB1 to suppress its function thereby activates YAP/TAZ.

### EGFR inhibition with erlotinib increases pYAP and suppresses transcription of YAP-regulated genes in cancer

**Fig. 5:**
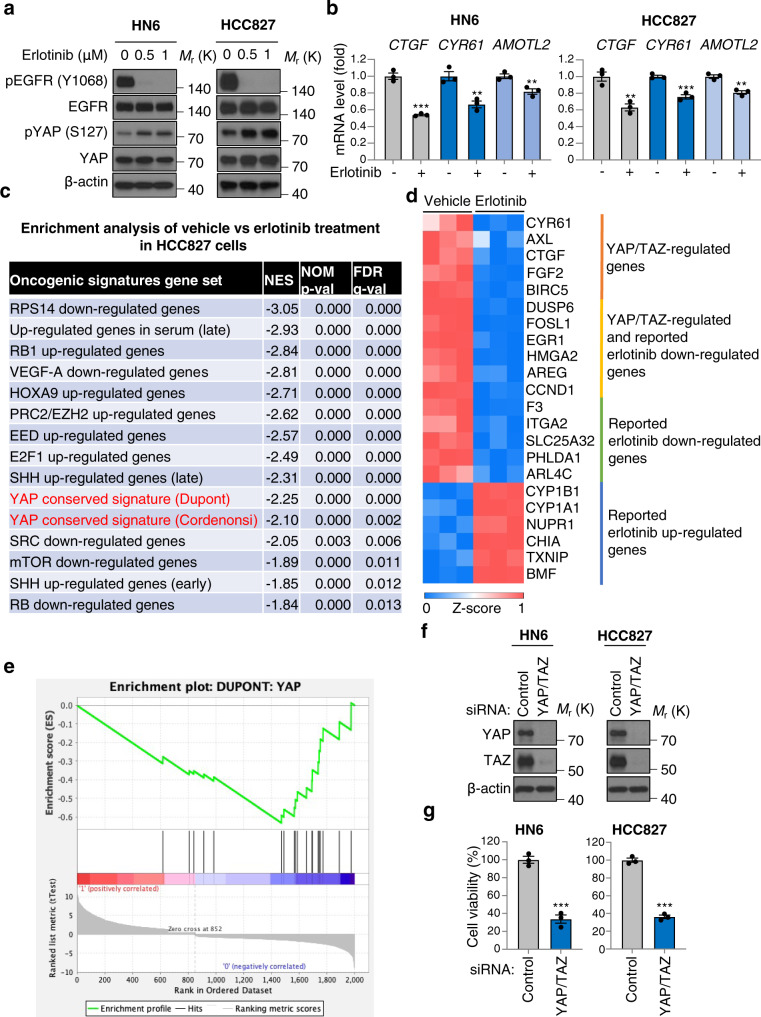
Erlotinib increases pYAP and suppresses YAP/TAZ-regulated genes. **a** Immunoblot of pEGFR (Y1068), EGFR, pYAP (S127), YAP, β-actin in WSU-HN6 and HCC827 cells. Cells were treated with erlotinib at the indicated concentrations for 2 h. **b** Relative mRNA levels of *CTGF*, *CYR61*, and *AMOTL2* in WSU-HN6 and HCC827 cells. Cells were treated with erlotinib (1 μM) for 2 h. **c** The top 15 enriched oncogenic signatures gene sets from RNA-seq data analysis of HCC827 cells. YAP-regulated signatures gene sets are highlighted in red. Cells were treated with vehicle or erlotinib (1 μM) for 24 h. The original name of signature gene sets are listed in supplementary Fig. S5a. **d** Heat map showing Z-score normalized mRNA expression of representative YAP/TAZ-regulated genes highlighted in orange and yellow. The genes highlighted in green and blue are consistent with the ones previously reported as up- or downregulated by erlotinib treatment^31^. **e** Enrichment plots of YAP conserved signatures. **f** Immunoblot of YAP, TAZ, β-actin in HN6 and HCC827 cells. Cells were transfected with siRNA for control and YAP/TAZ, and incubated for 48 h. **g** Cell viability. ANOVA with Tukey–Kramer post hoc test and Student’s *t*-test were used. Mean ± SEM (**b**, **g**); ***P <0.001; **P <0.01.

HNSCC is characterized by EGFR overexpression and amplification, while non-small cell lung cancer, especially lung adenocarcinoma, harbor frequent activating E746-A750 deletions or L858R mutations in *EGFR*^29,30^. Given this genetic background, we used HN6 showing the highest expression of EGFR among all HNSCC cell lines and HCC827 cells, lung adenocarcinoma cell lines harboring deletion of E746-A750 in EGFR. These cells were treated with erlotinib, an inhibitor of EGFR, and showed pYAP increase and suppression of *CTGF*, *CYR61*, and *AMOTL2* expression (Fig. 5a and b). To examine the comprehensive transcriptional changes of EGFR inhibition on a global level, we conducted mRNA-sequencing (RNA-seq) of HCC827 cells treated with vehicle or erlotinib, and performed differential gene expression analysis to identify genes whose expression levels were dysregulated in response to erlotinib treatment (Fig. 5c–e and Supplementary Fig. S5a–c). We observed that along with previously reported erlotinib-regulated genes, many genes that are known to be regulated by YAP/TAZ were also suppressed, including *CTGF*, *CYR61*, *AXL*, *FGF2*, *BIRC5*, *DUSP6*, *FOSL1*, *EGR1*, *HMGA2*, *AREG*, *CCND1* (Fig. 5d, and Supplementary Fig. S5b)^31^. To profile the transcriptional effects of inhibiting EGFR on a functional pathway level, we performed gene set enrichment analysis (GSEA) of the top dysregulated genes in response to erlotinib treatment, and found that YAP-regulated signatures gene sets (DUPONT: YAP and CORDENONSI_YAP_CONSERVED_SIGNATURE) were significantly downregulated in erlotinib treated cells (Fig. 5c–e, and Supplementary Fig. S5a–c)^32^. In addition, YAP/TAZ knockdown in HN6 and HCC827 cells significantly suppressed cell viability, consistent with their growth dependency on YAP/TAZ (Fig. 5f and g).

### Loss of LATS1/2 confers resistance to erlotinib in cancer cells with EGFR alterations

**Fig. 6:**
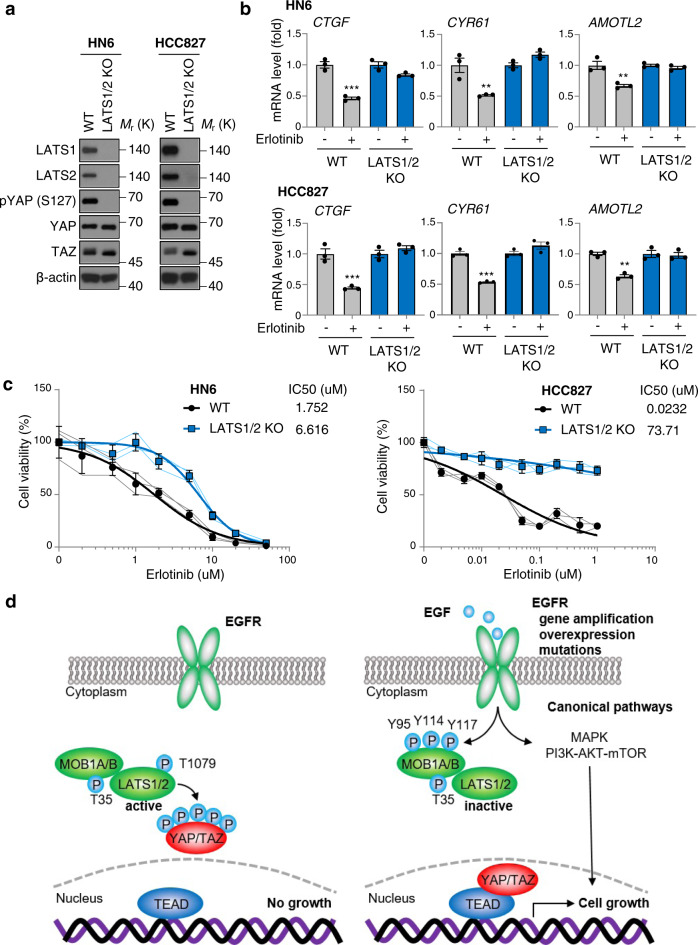
Loss of LATS1/2 confers resistance to erlotinib treatment in cancer cells with EGFR alterations. **a** Immunoblot of LATS1, LATS2, pYAP (S127), YAP, TAZ, β-actin in WT or LATS1/2 KO HN6 and HCC827 cells. **b** Relative mRNA levels of *CTGF*, *CYR61*, and *AMOTL2*. Cells were treated with erlotinib (1 μM) for 2 h. **c** Cell viability. Cells were treated with erlotinib for 3 days. **d** Schematic of EGFR-mediated YAP/TAZ activation. When EGFR is inactivated, the hydrophobic site of LATS1/2 is phosphorylated and LATS1/2 are active, leading to YAP/TAZ phosphorylation and cytoplasmic retention or degradation. Upon EGF stimulation or EGFR activation by gene amplification, overexpression or mutations, MOB1 is tyrosine phosphorylated and LATS1/2 are dephosphorylated and inactive, resulting in YAP/TAZ nuclear translocation and expression of growth promoting genes regulated by YAP/TAZ. ANOVA with Tukey–Kramer post hoc test were used. Mean ± SEM (**b**); ***P <0.001; **P <0.01. *versus WT erlotinib non-treated.

To examine the importance of YAP/TAZ activation under EGFR in HNSCC and lung adenocarcinoma cells, we genome edited the *LATS1* and *LATS2* genes to activate YAP/TAZ in both cells harboring EGFR alterations. Initially, we took advantage of the CRISPR/Cas9 system to knockout (KO) LATS1 in HN6 and HCC827 cells. LATS1 KO HCC827 cells showed resistance to erlotinib, while LATS1 KO HN6 failed to rescue proliferation, suggesting that LATS1 KO was not sufficient to induce YAP/TAZ activation (Supplementary Fig. S6a and b). Thus, we performed the additional knockdown of LATS2, which partially rescued erlotinib-inhibited *CTGF*, *CYR61*, and *AMOTL2* expression and conferred resistance to erlotinib (Supplementary Figs. S6c–f). As expected, the basal expression levels in LATS1/2 KO cells were much higher than those of LATS1 KO with siLATS2, suggesting complete knockout of LATS1/2 is required to fully activate YAP/TAZ (Supplementary Fig. S7a and b). We also confirmed that pYAP (S127) was completely dephosphorylated in LATS1/2 KO cells (Fig. 6a). Remarkably, LATS1/2 KO cells completely rescued erlotinib-inhibited *CTGF, CYR61, AMOTL2* expression and was sufficient to confer resistance to the growth suppressive effects of erlotinib (Fig. 6b and c). Both HN6 and HCC827 cells treated with erlotinib resulted in PARP cleavage, a typical molecular event caused by engagement of pro-apoptotic pathways. However, LATS1/2 KO cells showed a reduction in PARP cleavage, supporting that LATS1/2 deficiency confers resistance to erlotinib by promoting cell survival (Supplementary Fig. S7c and d).

### Loss of LATS1/2 confers resistance to erlotinib in cancer cells with EGFR alterations in vivo

**Fig. 7:**
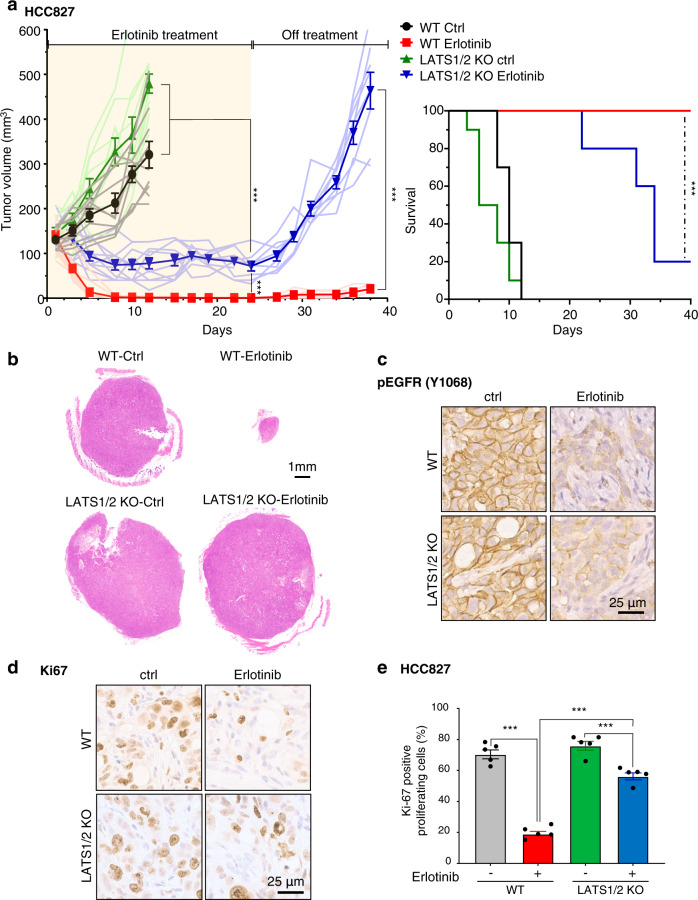
Loss of LATS1/2 confers resistance to erlotinib treatment in cancer cells with EGFR alterations in vivo. **a** (left) Individual and average (bold line) growth curves for HCC827 control and LATS1/2 KO cells transplanted into Female NOD-scid IL2Rgamma^null^ mice and treated with erlotinib for 24 days. (*n* = 10 per group). Tumor re-growth was monitored after erlotinib treatment was discontinued. (right) Kaplan–Meier curve showing survival of mice from (**a**). The death of animals occurred either naturally, when tumor growth compromised animal welfare, or when tumor volume reached >200% of initial size at day 1 of treatment. (*n* = 10 mice per group; Log-Rank/Mantel–Cox test.). **b** Representative histological sections from each treatment group. Scale bar represents 1 mm. **c**, **d** Representative immunohistochemical analysis of pEGFR and Ki67 in the short-term treatment groups (every day for 5 days). **e** Quantification of (**d**) showing percentage of cells staining positive for Ki67 (*n* = 5 mice per group). Mean±SEM (**a**, **e**); ***P < 0.001.

To further investigate the role of YAP/TAZ activation as a downstream signal of EGFR in HNSCC, we implanted WT and LATS1/2 KO HCC827 cells into NOD-SCID mice, and treated them with erlotinib or vehicle control. While the WT group showed remarkable reduction in tumor volume in response to erlotinib treatment and did not show regrowth after achieving near complete responses, the LATS1/2 KO group exhibited a significant but more limited tumor growth reduction during erlotinib treatment, and rapid regrowth after cessation of erlotinib administration (Fig. 7a(left), b). In line with the tumor growth curves, the LATS1/2 KO group demonstrated a beneficial response to erlotinib treatment, but a significantly poorer survival compared to WT tumors (Fig. 7a(right)). Immunohistochemical analysis showed that pEGFR was suppressed in both WT and LATS1/2 groups, and that the percentage of Ki67 positive proliferating cells in erlotinib-treated LATS1/2 group was significantly higher than that of erlotinib-treated WT group (Fig. 7c–e). These results indicate that YAP/TAZ activation may underlie intrinsic as well as acquired resistance to EGFR inhibition in EGFR-altered cancer cells, as judged by reduced tumor growth suppression and rapid tumor relapse.

## Discussion

Here, we demonstrate that activated EGFR due to overexpression, amplification or mutations induces YAP/TAZ activation. In the absence of growth factor stimulation, Hippo pathway activity results in LATS1/2 phosphorylation in its hydrophobic motif (T1079), leading to high kinase activity and the phosphorylation, cytoplasmic retention and/or degradation of YAP/TAZ (Fig. 6d). Upon EGFR activation by ligand exposure, gene amplification, overexpression, or mutation, EGFR promotes the phosphorylation of MOB1 at Y95, Y114, and Y117 resulting in reduced LATS1/2 phosphorylation and function, and the consequent YAP/TAZ hypo-phosphorylation, nuclear translocation, interaction with TEADs, and activation of growth-promoting transcriptional networks (Fig. 6d).

Interestingly, EGFR stimulation results in the rapid reduction of YAP phosphorylation, preceding the increase in CTGF, CYR61, and AMOTL2 expression at the protein and mRNA level that lasts several hours. This suggests that transcription of YAP/TAZ-regulated genes is initiated rapidly, and that other transcription factors downstream of EGFR may subsequently sustain their expression. Aligned with this possibility, while EGFR activation by EGF treatment strongly induced transcription of *CTGF* and *CYR61*, it was diminished by YAP/TAZ knockdown albeit it did not completely suppress *CYR61* elevation although *CTGF* was clearly inhibited. Emerging evidence suggest that various transcription factors such as AP-1 and chromatin remodeling molecules, including the BRD4 and SWI/SNF complexes, regulate YAP/TAZ/TEAD and synergistically increase or suppress their target gene transcription^33–35^. Especially, activator protein-1 (AP-1, which primarily includes dimer of JUN and FOS proteins), act downstream of ERK1/2^36^. In response to the activation by the EGFR-MAPK pathway, AP-1 complexes co-occupied distal enhancers with YAP/TAZ/TEADs, contacting YAP/TAZ-target gene promoters through chromatin loops^33,37^. Therefore, sustained and prolonged expression may involve AP-1 activation by EGFR-MAPK, in addition to YAP/TAZ.

Although the effector of the Hippo pathway, YAP was initially identified as a substrate of YES and other Src-family kinases, the role of tyrosine phosphorylation of the core Hippo pathway components has not been studied in detail as compared to the large body of information regarding the regulation of this pathway by serine/threonine protein phosphorylation^38–40^. However, recent findings support that the Hippo pathway can be regulated by tyrosine kinases. For example, FGFR4 and c-Abl phosphorylate MST1 at Y433^25,26^, and Src can phosphorylate LATS1^27^. Of importance, our recent work has shown that MOB1 is phosphorylated by FAK at Y26, leading to dissociation of LATS1/2 and MOB1 for YAP activation^28^. Our current study revealed that Y95, Y114, Y117 of MOB1 could be phosphorylated downstream from EGFR. While EGFR can phosphorylates MOB1 in vitro, it is possible that intermediate receptor or non-receptor tyrosine kinases can contribute to the EGFR-induced phosphorylation. Aligned with our results, a phosphoproteomic dataset of PC-9 lung adenocarcinoma cells harboring active EGFR via E746-A750 activating deletions (PhosphoSitePlus; https://www.phosphosite.org/uniprotAccAction?id=Q9H8S9), showed elevated levels of MOB1 phosphorylated at Y95, supporting our findings that EGFR-MOB1-YAP/TAZ signaling may play an important role in cancers harboring EGFR alterations^41,42^.

Distinct from FAK-induced phosphorylation of MOB1 on Y26 and its dissociation from LATS^28^, however, EGFR activation did not affect the interaction between MOB1 and LATS1/2, while it induced hypo-phosphorylation of the hydrophobic motif (T1079) in LATS1/2 and suppressed their kinase activity. The hydrophobic motif of LATS1/2 can be phosphorylated by activated MST1/2, although EGFR did not change the activity of MST1/2. Similar to MST1/2, mitogen-activated protein kinase kinase kinase kinase (MAP4K) family members, TAOK1 and TAOK3 are also capable of phosphorylating the hydrophobic motif of LATS1/2^43–46^. Given that the hydrophobic motif of LATS1 is under-phosphorylated upon EGFR activation and LATS1 phosphorylation remains higher when MOB1 tyrosine phospho acceptor sites are mutated, it is possible that conformational changes triggered by tyrosine-phosphorylation interfere with the interaction between LATS1/2 and MST1/2, MAP4Ks, or TAOKs. Further studies, including structural analysis, will be required to clarify the precise role of MOB1 tyrosine phosphorylation in LATS1/2 regulation, including the possibility that this may result in the interaction of the MOB1/LATS1/2 complex with other upstream components or regulatory molecules. Notably, the distinct regulation of MOB1 tyrosine phosphorylation likely represents a previously unappreciated regulatory signaling node by which multiple receptor and non-receptor tyrosine kinases may converge with the Hippo pathway to control YAP/TAZ activity

Tyrosine kinase inhibitors (TKIs) are well accepted as a molecular targeted therapies for patients with cancers harboring EGFR mutations. EGFR-TKIs including erlotinib and gefitinib (first-generation reversible), afatinib (second-generation irreversible), osimertinib (third-generation irreversible) have been approved for the treatment of lung cancer patients harboring EGFR mutations^47–50^, and cetuximab has been used for HNSCC and colorectal cancer patients^19,20,51^. While cetuximab is the only FDA-approved cancer-targeting drug for patients with HNSCC, monotherapy response rate is limited (only 10–30%), suggesting the possibility of intrinsic or acquired resistance^29^. Similarly, use of EGFR TKIs in lung cancer show improved response rates (50–80%), but the emergence of intrinsic or acquired resistance, for example, the emergence of EGFR-T790M mutations or the activation of other signaling pathways including MET, AXL, IGF1R, IL-6R, HER2, and HER3^52^, often results in tumor relapse and progressive disease. Of importance, emerging evidence have shown that YAP is overexpressed and contributes to acquired resistance and poor prognosis of cetuximab in HNSCC or EGFR TKIs in lung cancers^52–56^. These prior reports in conjunction with our findings altogether support the theory that a prevalent mechanism of resistance to EGFR-targeted therapies is through the re-activation of YAP/TAZ. Loss of LATS1/2 or other Hippo pathway alterations could confer resistance to erlotinib in HNSCC cells with EGFR overexpression or lung adenocarcinoma cells harboring EGFR mutations. Specifically, our in vivo experiments showing a significant but more limited response to erlotinib in LATS1/2 KO group and rapid tumor regrowth after erlotinib treatment support the idea that YAP/TAZ activation plays an important role in therapy resistance and in tumor recurrence. Therefore, YAP and TAZ may represent mechanistic therapeutic targets in combination with EGFR targeting therapy in order to prevent cancer cells from acquiring resistance and the consequent treatment failure.

Taken together, our study revealed that EGFR promotes MOB1 phosphorylation and suppresses the Hippo pathway, leading to aberrant YAP/TAZ activation in cancers harboring EGFR alterations. These findings support that the EGFR-MOB1-YAP/TAZ signaling axis may represent a novel therapeutic target for preventing cancer recurrence and progression.

## Methods

### Antibodies and reagents

Anti-pEGFR (Y1068) (#2234, 1:5000), EGFR (#4267, 1:5000), pYAP (S127) (#4911, 1:2000), YAP (#14074, 1:1000), YAP/TAZ (#8418, 1:1000), pLATS1 (T1079) (#8654, 1:1000), LATS1 (#3477, 1:1000), LATS2 (#5888, 1:1000), pMST1/2 (T183/180) (#3681, 1:1000), MST1 (#3682, 1:1000), pMOB1 (T35) (#8699, 1:1000), MOB1 (#13730, 1:1000), TEAD1 (#12292, 1:1000), HA-tag (#3724, 1:10000), Myc-tag (#2276, 1:5000), FLAG-tag (#2368, 1:5000), GST-tag (#2624, 1:10000), pTyrosine (P-Y-100) (#9411, 1:2000), CTGF (#86641, 1:1000), CYR61 (#14479, 1:1000), pERK1/2 (T202/Y204) (#4370, 1:10000), ERK1/2 (#4696, 1:10000), pAKT (S473) (#4060, 1:5000), AKT (#9272, 1:5000), pS6 (S235/236) (#4858, 1:10000), S6 (#2217, 1:10000), PARP (#9542, 1:1000), GRB2 (#36344, 1:1000), β-actin (#4967, 1:5000) were purchased from Cell Signaling Technology (MA).

EGF (#E9644) was purchased from Sigma-Aldrich Inc (MO). BYL719 (#16986) was purchased from Cayman Chemical (MI).

### Cell culture and transfection

CAL33, CAL27, and HN6 cells were obtained from the NIDCR Oral and Pharyngeal Cancer Branch cell collection^21^. Their identity was confirmed by STR profiling and they were tested free of mycoplasma infection. HEK293 cells were purchased from ATCC (Manassas, VA). CAL33, CAL27, HN6, and HEK293 cells were cultured in DMEM (D-6429, Sigma-Aldrich Inc., St. Louis, MO) supplemented with 10% FBS (Sigma-Aldrich Inc., St. Louis, MO), 1× antibiotic/antimycotic solution (Sigma-Aldrich Inc., MO) and 5 μg/ml plasmocin^TM^ prophylactic (InvivoGen, CA). HCC827 cells were purchased from ATCC and cultured in RPMI 1640 medium, GlutaMAX^TM^ supplement (#61870-036, Thermo Fisher Scientific, CO).

### CRISPR/Cas9 genome editing

pLentiCRISPRv2 expressing CAS9 and sgRNA against *LATS1* and *LATS2* were purchased from Genscript (*LATS1*: guide RNA1, *LATS2*: guide RNA1). Lentivirus was produced in HEK293 cells, lentiviral supernatant was filtered through a 0.45-μm syringe filter, then infected with polybrene (10 μg/ml, Sigma-Aldrich Inc., MO). Infected cells were cultured and selected in the presence of puromycin for 7 days.

### Western blotting and immunoprecipitation

For western blotting, cells were harvested after 2 times rinse by cold PBS, lysed in RIPA buffer (50 mM Tris-HCl, 150 mM NaCl, 1 mM EDTA, 1% NP-40) supplemented with Halt^TM^ Protease and Phosphatase Inhibitor Cocktail (#78440, ThermoFisher Scientific). Lysate was sonicated 3 times for 5 s, incubated for 15 min on ice, then centrifuged for 15 min. The concentration of supernatants was measured. Equal amounts of protein were loaded for SDS-PAGE, and transferred to PVDF membranes. The membranes were blocked with 3% BSA in TBS-T buffer for 20 min. Then, the membranes were incubated with primary antibodies diluted by 3% BSA or 5% non-fat milk in TBS-T buffer for 2 h at room temperature. After washing by TBS-T buffer 3 times, the membranes were incubated with secondary antibodies (HRP-conjugated goat anti-mouse or anti-rabbit IgG at 1:20,000 dilution, Southern Biothech) diluted by 5% non-fat milk in TBS-T buffer for 1 h at room temperature. Immobilon Western Chemiluminescent HRP substrate (Millipore, MA) was used for detection. For immunoprecipitation, cells were lysed in CHAPS buffer (1% CHAPS, 30 mM Tris-HCl, 150 mM NaCl) with protease and phosphatase inhibitor (#78444 Thermo Fisher Scientific, CO) and 1 mM DTT, incubated on ice for 15 min, and centrifuged at 16,000 × *g* for 15 min at 4 °C. Supernatants were incubated primary antibody for 24 h at 4 °C, then incubated with protein G or A agarose beads for 1 h at 4 °C. Beads were centrifuged and rinsed with CHAPS buffer 5 times, then boiled with sample buffer. Beads were centrifuged and the supernatants were used for western blotting. Image J software was used for densitometry analysis of the bands. Uncropped blots are shown in Supplementary Figs. S8–21.

### DNA constructs

Plasmids pCMV-myc-MST1 (Addgene #8847 from Joseph Avruch’s lab)^57^, pCMV2-FLAG-SAV1 (Addgene #18970 from Marius Sudol’s lab)^58^, pcDNA3-HA-MOB1 (Addgene #32835 from Kunliang Guan’s lab)^14^, p2xFLAG-CMV2-LATS1 (Addgene #18971 from Marius Sudol’s lab)^58^, and pQCXIH-Myc-YAP (Addgene #33091 from Kunliang Guan’s lab)^14^ were used. GFP-expressing vector was used as control.

### Preparation of recombinant protein

GST-MOB1 was subcloned from HA-MOB1 plasmid into pGEX4T3 vector. pGEX-MOB1 WT was transformed in escherichia coli BL21. E. coli containing pGEX-MOB1 WT were cultured at 37 °C for 3 hr, then cultured with 1 mM isopropyl-β-D-thiogalactopyranoside (IPTG) at 25 °C overnight. The proteins purification step was performed using magneGST^TM^ protein purification system, following the manufacturer’s protocol (Promega, WI).

### In vitro kinase assay

Recombinant EGFR (#3641, Sigma-Aldrich, MO), purified recombinant GST-MOB1 protein, 200 μM ATP (#9804, Cell Signaling Technology, MA), 1× kinase buffer (#9802, Cell Signaling Technology, MA) were mixed and incubated at 30 °C for 30 min. The reaction was stopped by adding sample buffer, and incubated at 95 °C for 5 min. For LATS1 kinase activity, HEK293 cells were harvested and used for immunoprecipitation as described above. Primary antibody against IgG or LATS1 were used. After washing the beads, the beads were incubated with recombinant YAP (ab132459, Abcam, CA), 200 μM ATP (#9804, Cell Signaling Technology, MA), 1× kinase buffer (#9802, Cell Signaling Technology, MA) at 30 °C for 30 min. The reaction was stopped by adding sample buffer, then incubated at 95 °C for 5 min. The beads were centrifuged and removed, and supernatant was used for western blot.

### Knockdown by siRNA

Cells were transfected with siRNA using Lipofectamine RNAiMAX Reagent (Thermo Fisher Scientific). The sequence of siRNA for YAP (SMARTpool siGENOME YAP1, #M-012200-00-0005) was purchased from Dharmacon (CO), TAZ (Silencer Select siRNA for WWTR1, #4427037) was from Thermo Fisher Scientific (CO), LATS1 (Hs01_00046130), LATS2 #1 (Hs01_00158804) and negative control (SIC001) were from Sigma-Aldrich, (MO).

### MOB1 point mutation

MOB1 8YF, 7YF + Y, and 3YF were generated using the QuikChange Lightning Site-Directed Mutagenesis Kit, following the manufacturer’s protocol (Agilent Genomics, CA). pcDNA3-HA-MOB1 was used as template for mutagenesis. All mutated sites were validated by sequencing.

### Cell viability assay

Cells were plated on 96 well plates. After cells attached on the plate, cells were treated with reagent for 3 days. Aquabluer reagent (#6015, MutliTarget Pharmaceuticals LLC, CO) was applied in the culture medium, incubated for 2 h, then the absorbances were measured by a microplate reader.

### RNA isolation and real-time PCR

RNA was extracted using RNeasy Mini Kit following the manufacturer’s instruction (#74104, Qiagen, Hilden, Germany). Five hundred nanogram of total RNA was used for cDNA synthesis using SupreScript^TM^ VILO^TM^ cDNA Synthesis Kit (#11754250, Thermo Fisher Scientific, CO). Real-time PCR was performed using SYBR^TM^ Select Master Mix (#4472908, Thermo Fisher Scientific, CO). The following primers were used. GAPDH F: 5′-GAGTCAACGGATTTGGTCGT-3′, R: TTGATTTTGGAGGGATCTCG-3′, CTGF F: 5′-GTTTGGCCCAGACCCAACTA-3′, R: GGCTCTGCTTCTCTAGCCTG-3′, CYR61 F: 5′-CAGGACTGTGAAGATGCGGT-3′, R: GCCTGTAGAAGGGAAACGCT-3′, and AMOTL2 F: 5′-AGCTTCAATGAGGGTCTGCT-3′, R: 5′-TGAAGGACCTTGATCACTGC-3′.

### Immunofluorescence

Cells were cultured on coverslips coated with Poly-D-lysine hydrobromide (#P7280, Sigma-Aldrich Inc., MO) were rinsed with PBS, fixed with 4 % paraformaldehyde in PBS for 30 min, and permeabilized using 0.5% Triton X-100 with 200 mM glycine for 10 min. Fixed cells were blocked with 3% BSA-containing PBS for 1 h at room temperature, and incubated with primary antibody overnight at 4 °C. Then, incubated with alexa-labeled secondary antibodies (Goat anti-Rabbit IgG Alexa Fluor 488, #A11008, Thermo Fisher Scientific, CO) for 1.5 h at room temperature. Cells were stained with DAPI (#GTX16262, GeneTex, CA) for 10 min at room temperature and mounted. Images were acquired with Zeiss LSM 880 with Airyscan (Carl Zeiss, NY).

### RNA sequencing

Samples were sequenced using the Illumina platform. For each sample, paired end sequencing reads were mapped using Bowtie2 version 2.3.4 to GRCh38 reference human genome downloaded from Ensembl. To compute transcript abundance, uniquely mapped reads were quantified using featureCounts version 1.6.3. Counts tables were uploaded to the Galaxy web platform and using the public server at usegalaxy.org, EntrezIDs were converted to gene symbols using the annotatemy IDs tool^59^. Differential gene expression analysis was performed using DESeq2 version 1.18.1, using parametric fit.

### Gene set enrichment analysis (GSEA)

For the analysis of CCLE data^22^, GSEA (Broad Institute, http://software.broadinstitute.org/gsea/index.jsp) was performed using with 1000 permutations, “Pearson” metric of RNA-seq read counts per gene and a gene set size filter of 15-500. The “C6” gene set database from MSigDB (Cordenonsi: YAP conserved signature”) was spiked with “DUPONT: YAP”^60^ and “ZHAO: Induced_by_YAP”^61^. For the analysis of the RNAseq data, GSEA was performed the same as above, using a *t*-test metric for ranking genes.

### In vivo mouse experiments

All the animal studies using tumor xenografts studies were carried out according to the UCSD approved protocol (ASP # S15195) in compliance with the IACUC Guide for the Care and Use of Laboratory Mice. Female NOD-scid IL2Rgamma^null^ mice (4–6 weeks of age) were purchased from Charles River Laboratories (Worcester, MA, USA). Cells were transplanted into both flanks (2 million per tumor) of each mouse. When average tumor volume reached a predetermined volume (~150 mm^3^) the mice were randomized into groups (10 tumors per group). For drug treatment, the mice were treated (oral gavage) with erlotinib (Selleck Chemicals, 50 mg/kg/day) or control diluent (15% Captisol). The mice were euthanized at the indicated time points, when mice succumbed to disease, when tumor growth compromised animal welfare, or when tumor volume reached >200% of initial size at day 1 of treatment. Tumors were isolated for histologic and immunohistochemical evaluation.

### Immunohistochemistry

All tissue samples were processed and stained as previously described^62^. The following antibodies were used: pEGFR (catalog number API300AA) was from Biocare Medical (Pacheco, CA, USA). Ki67 (catalog number ab15580) was from Abcam (Cambridge, MA, USA). Samples were scanned with Axioscan Z1 (Zeiss).

### Statistics and reproducibility

All data were analyzed using GraphPad Prism version 7 for Windows (GraphPad Software, CA). The data were analyzed by Student’s *t*-test (two-sided) and ANOVA with Tukey–Kramer post hoc test or as indicated in figure legends. All experiments were repeated independently at least three times with similar results, with the exception of the animal studies that were conducted once with a large cohort of mice.

## Supplementary information

Supplementary Information

Description of Additional Supplementary Files

Supplementary Data 1

## Data Availability

CCLE data set is available online (https://portals.broadinstitute.org/ccle). TCGA data is available from c-bioportal (https://www.cbioportal.org/). All other data that support the findings of this study are available from the corresponding author upon reasonable request. The source data underlying Figs. 1b, e, 2b, d, 4f, 5b, g, 6b, c, 7e and Supplementary Figs S3c, S4c, f, g, S6a-d, f, S7a, b are provided as a Supplementary Data 1. Raw and processed RNAseq data have been deposited in NCBI’s Gene Expression Omnibus (GEO) and can be accessed under GEO Series Accession Number GSE178755.
