# Supplementary material for: EGFR Regulates the Hippo pathway by promoting the tyrosine phosphorylation of MOB1
Source: Commun Biol. 2021 Nov 1;4:1237. doi: 10.1038/s42003-021-02744-4 (PMC8560880; doi:10.1038/s42003-021-02744-4)
Supplement: Supplementary file 1 — Supplementary Information [file 42003_2021_2744_MOESM1_ESM.pdf]

Supplementary Information

Rank	Oncogenic signatures gene set	NES	NOM p-val	FDR q-val
1	EGFR_UP.V1_UP	2.27	0.00	0.00
2	MEK_UP.V1_UP	2.27	0.00	0.00
3	P53_DN.V1_UP	2.22	0.00	0.00
4	RAF_UP.V1_UP	2.14	0.00	0.0015
5	ESC_V6.5_UP_EARLY.V1_DN	2.13	0.00	0.0015
6	AKT_UP.V1_UP	2.12	0.00	0.0013
7	RB_DN.V1_DN	2.10	0.00	0.0014
8	BMI1_DN.V1_UP	2.09	0.00	0.0014
9	ESC_J1_UP_LATE.V1_UP	2.08	0.00	0.0016
10	PTEN_DN.V2_UP	2.07	0.00	0.0015
11	TGFB_UP.V1_UP	2.04	0.00	0.0022
12	ERB2_UP.V1_UP	2.03	0.00	0.0029
13	MEL18_DN.V1_UP	2.03	0.0060	0.0028
14	AKT_UP_MTOR_DN.V1_UP	2.01	0.00	0.0033
15	BMI1_DN_MEL18_DN.V1_UP	2.00	0.0083	0.0037
16	CRX_DN.V1_DN	1.98	0.00	0.0038
17	TBK1.DF_UP	1.95	0.00	0.0052
18	CAHOY_ASTROGLIAL	1.94	0.00	0.0057
19	MYC_UP.V1_DN	1.92	0.0020	0.0070
20	STK33_NOMO_UP	1.91	0.00	0.0074
21	DUPONT: YAP	1.91	0.017	0.0070
22	CORDENONSI_YAP_CONSERVED_SIGNATURE	1.88	0.019	0.0091
23	E2F1_UP.V1_DN	1.84	0.0021	0.013
24	KRAS.DF.V1_UP	1.83	0.0082	0.015
25	PRC2_EZH2_UP.V1_UP	1.83	0.0021	0.015
26	GCNP_SHH_UP_LATE.V1_DN	1.82	0.0020	0.015
27	SNF5_DN.V1_DN	1.82	0.0020	0.014
28	ZHAO: INDUCED BY YAP	1.81	0.0041	0.015
29	STK33_UP	1.80	0.0040	0.017
30	LTE2_UP.V1_DN	1.78	0.0079	0.019

Supplementary Figure S1

The top 30 enriched oncogenic signatures gene sets with EGFR expression in CCLE data, related to figure 1c. YAP-regulated gene signatures gene sets are highlighted in red.

a

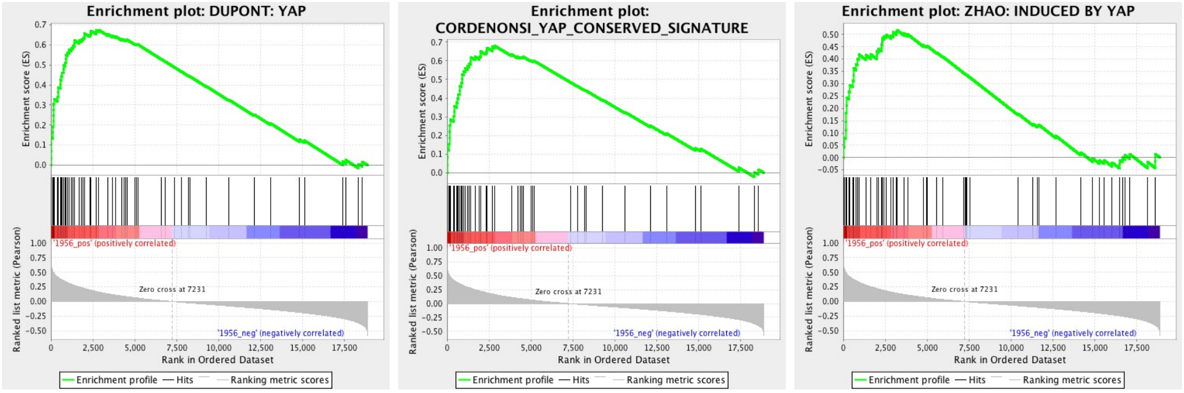

b

DUPONT: YAP

	GENE SYMBOL	RANK METRIC SCORE	RUNNING ES
1	AMOTL2	0.628832	0.034931
2	CYR61	0.623759	0.070211
3	ARHGAP29	0.609183	0.104187
4	CRIM1	0.597951	0.13795
5	SH2D4A	0.519249	0.162836
6	MYO1C	0.514652	0.191661
7	AXL	0.506905	0.220153
8	THBS1	0.506506	0.248835
9	ANLN	0.496296	0.276247
10	ITGB5	0.493865	0.303786
11	SERPINE1	0.486336	0.33042
12	DUSP1	0.418719	0.342159
13	TGFB2	0.414801	0.364682
14	TGM2	0.413047	0.387477
15	GADD45B	0.388605	0.403356
16	GLIS2	0.385049	0.423818
17	FSTL1	0.377259	0.442722
18	FLNA	0.3707	0.461626
19	GAS6	0.360303	0.478505
20	CTGF	0.35532	0.496961

CORDENONSI_YAP_ CONSERVED_SIGNATURE

	GENE SYMBOL	RANK METRIC SCORE	RUNNING ES
1	AMOTL2	0.628831744	0.041748118
2	CYR61	0.623759389	0.08379114
3	CRIM1	0.597951233	0.12339943
4	SH2D4A	0.519249082	0.1539168
5	AXL	0.506904781	0.18747908
6	THBS1	0.506505668	0.22165206
7	ITGB5	0.493865341	0.25374871
8	SERPINE1	0.486335635	0.28565538
9	DUSP1	0.418718696	0.30194032
10	TGFB2	0.4148013	0.3289601
11	TGM2	0.413046807	0.35623324
12	GADD45B	0.388604522	0.37632844
13	FSTL1	0.377258956	0.39788875
14	FLNA	0.370700061	0.42081255
15	GAS6	0.360302627	0.44159904
16	CTGF	0.355320245	0.4639087
17	HEXB	0.341654003	0.482691
18	DDAH1	0.336581469	0.503522
19	NDRG1	0.33564648	0.525618
20	FSCN1	0.324788004	0.542995

ZHAO: INDUCED_BY_YAP

	GENE SYMBOL	RANK METRIC SCORE	RUNNING ES
1	CYR61	0.623759	0.042776
2	PRSS23	0.570706	0.081092
3	AXL	0.506905	0.112124
4	CXCL2	0.494111	0.145555
5	ABCC3	0.49162	0.17945
6	SERPINE1	0.486336	0.212922
7	CCND1	0.469547	0.243676
8	LCN2	0.436312	0.268542
9	ALCAM	0.420637	0.294436
10	MICALL2	0.388551	0.313088
11	NFKBIZ	0.378789	0.336105
12	INHBA	0.378053	0.362472
13	CTGF	0.35532	0.3797
14	PTGS2	0.344929	0.400452
15	HMGA2	0.333162	0.41953
16	FST	0.284111	0.417154
17	EGR1	0.253483	0.418481
18	FGF2	0.22249	0.41583
19	FLRT2	0.222184	0.431229
20	WISP2	0.216385	0.441811

Supplementary Figure S2

(a) Enrichment plots (upper). (b) Top 20 genes with rank metric score and running enrichment score (ES) in three YAP-regulated gene signatures gene sets, related to figure 1c.

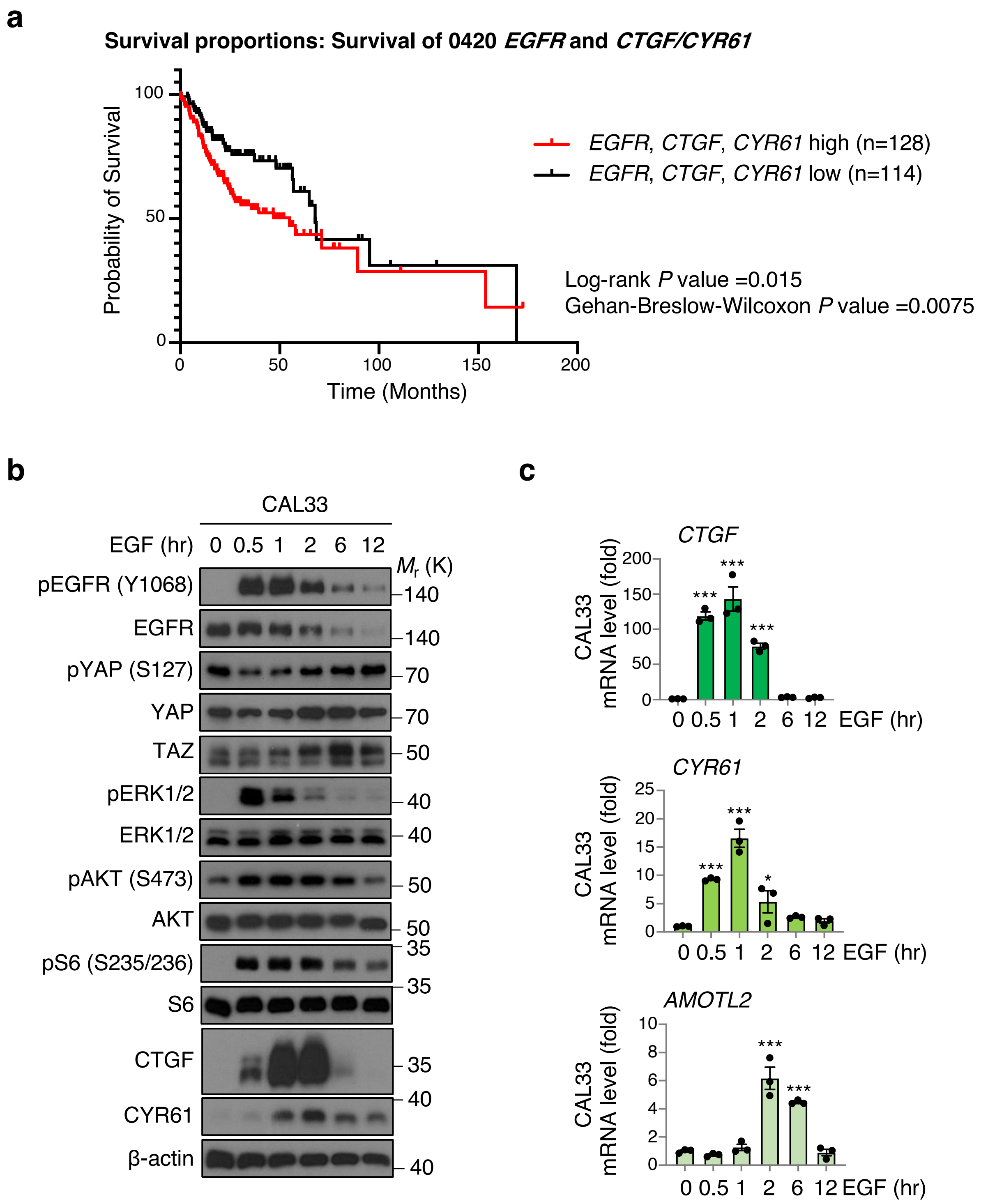
a
Survival proportions: Survival of 0420 EGFR and CTGF/CYR61
Probability of Survival
Time (Months)
EGFR, CTGF, CYR61 high (n=128)
EGFR, CTGF, CYR61 low (n=114)
Log-rank P value =0.015
Gehan-Breslow-Wilcoxon P value =0.0075
b
CAL33
EGF (hr) 0 0.5 1 2 6 12
Mr (K)
pEGFR (Y1068)
EGFR
pYAP (S127)
YAP
TAZ
pERK1/2
ERK1/2
pAKT (S473)
AKT
pS6 (S235/236)
S6
CTGF
CYR61
β-actin
c
CTGF
CYR61
AMOTL2
CAL33 mRNA level (fold)
EGF (hr)

Supplementary Figure S3

(**a**) Overall survival rate for HNSCC patient comparing 2 groups; EGFR, CTGF, CYR61 high group (EGFR>0, CTGF>0, CYR61>0, n=128) and EGFR, CTGF, CYR61 low group (EGFR<0, CTGF<0, CYR61<0, n=114), related to figure 1a-c. Data from TCGA (n=526) was analyzed. Log-rank (Mantel-Cox) test shows P=0.015, and Gehan-Breslow-Wilcoxon test shows P=0.0075. (**b**) Immunoblot of pEGFR (Y1068), EGFR, pYAP (S127), YAP, TAZ, pERK1/2 (T202/Y204), ERK1/2, pAKT (S473), AKT, pS6 (S235/236), S6, CTGF, CYR61, β-actin in CAL33 cells. Cells were serum starved for 16 hr, and treated with EGF (20 ng/ml) for the indicated time, related to figure 1d and e. (**c**) Relative mRNA levels of *CTGF*, *CYR61* and *AMOTL2*, related to figure 1d and e. ANOVA with Tukey-Kramer post hoc test were used. Mean±SEM (**c**); ***, $P < 0.001$; *, $P < 0.05$. *versus EGF 0 hr.

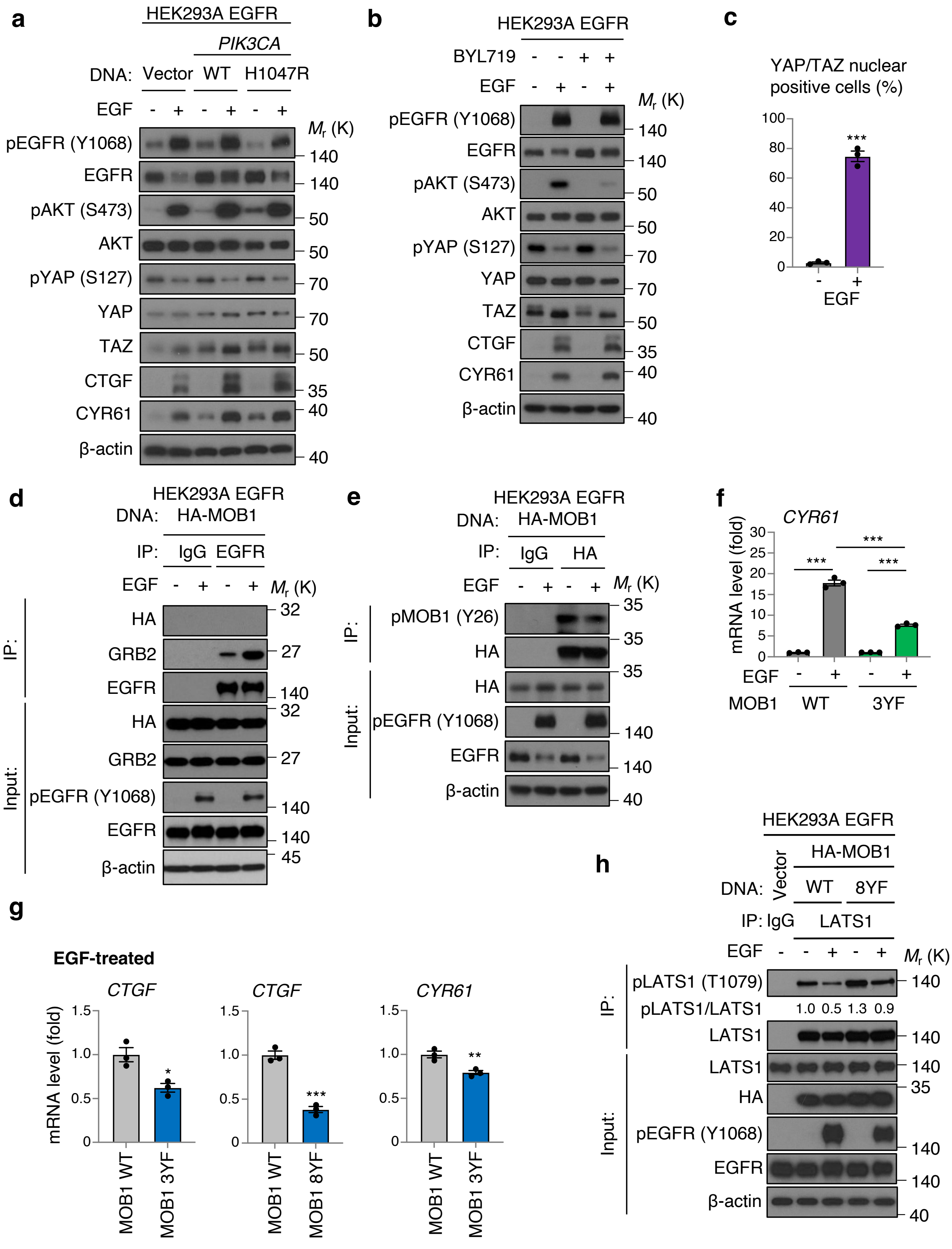
a
HEK293A EGFR
PIK3CA
DNA: Vector WT H1047R
EGF - + - + - +
M_r (K)
pEGFR (Y1068) 140
EGFR 140
pAKT (S473) 50
AKT 50
pYAP (S127) 70
YAP 70
TAZ 50
CTGF 35
CYR61 40
β-actin 40
b
HEK293A EGFR
BYL719 - - + +
EGF - + - +
M_r (K)
pEGFR (Y1068) 140
EGFR 140
pAKT (S473) 50
AKT 50
pYAP (S127) 70
YAP 70
TAZ 50
CTGF 35
CYR61 40
β-actin 40
c
YAP/TAZ nuclear positive cells (%)
100 80 60 40 20 0
***
- +
EGF
d
HEK293A EGFR
DNA: HA-MOB1
IP: IgG EGFR
EGF - + - +
M_r (K)
IP:
HA 32
GRB2 27
EGFR 140
Input:
HA 32
GRB2 27
pEGFR (Y1068) 140
EGFR 140
β-actin 45
e
HEK293A EGFR
DNA: HA-MOB1
IP: IgG HA
EGF - + - +
M_r (K)
IP:
pMOB1 (Y26) 35
HA 35
Input:
HA 35
pEGFR (Y1068) 140
EGFR 140
β-actin 40
f
CYR61
mRNA level (fold)
30 25 20 15 10 5 0
*** *** ***
EGF - + - +
MOB1 WT 3YF
g
EGF-treated
CTGF CTGF CYR61
mRNA level (fold)
1.5 1.0 0.5 0
* *** **
MOB1 WT MOB1 3YF
MOB1 WT MOB1 8YF
MOB1 WT MOB1 3YF
h
HEK293A EGFR
Vector HA-MOB1
DNA: WT 8YF
IP: IgG LATS1
EGF - - + - +
M_r (K)
IP:
pLATS1 (T1079) 140
pLATS1/LATS1 1.0 0.5 1.3 0.9
LATS1 140
Input:
LATS1 140
HA 35
pEGFR (Y1068) 140
EGFR 140
β-actin 40

Supplementary Figure S4

(**a**) Immunoblot of pEGFR (Y1068), EGFR, pAKT (S473), AKT, pYAP (S127), YAP, TAZ, CTGF, CYR61, β-actin in stably overexpressing vector, *PIK3CA* WT or H1047R HEK293A cells. Cells were transfected with EGFR plasmid and incubated for 24 hr, serum starved for 16 hr, and treated with EGF (20 ng/ml) for 1 hr, related to figure 2a. (**b**) Immunoblot of pEGFR (Y1068), EGFR, pAKT (S473), AKT, pYAP (S127), YAP, TAZ, CTGF, CYR61, β-actin in EGFR-overexpressing HEK293A cells. Cells were serum starved for 16 hr, and pretreated with BYL719 (1 μM) for 1 hr and followed by EGF treatment (20 ng/ml) for 1 hr, related to figure 2a. (**c**) Quantitative analysis of YAP/TAZ nuclear positive cells (%) of figure 2f. (**d**) Co-immunoprecipitation of HA-MOB1 and EGFR/GRB2. Lysates were immunoprecipitated with control IgG and an antibody against EGFR. Immunoblot of EGFR, GRB2, HA, pEGFR (Y1068), β-actin. EGFR-overexpressing HEK293A cells were transfected with HA-MOB1 plasmid and incubated for 24 hr, serum starved for 16 hr, and treated with EGF (20 ng/ml) for 5 min, related to figure 3d. (**e**) Immunoprecipitation of HA-MOB1. Lysates were immunoprecipitated with control IgG or antibodies against HA-tag. Immunoblot of pMOB (Y26), HA, pEGFR (Y1068), EGFR, β-actin. EGFR-overexpressing HEK293A cells were transfected with HA-MOB1 plasmid, incubated for 24 hr, serum starved for 16 hr, and treated with EGF (20 ng/ml) for 1 hr, related to figure 3d. (**f**) Relative mRNA levels of *CYR61* in EGFR-overexpressing HEK293A cells. Cells were transfected with MOB1 WT and 3YF, incubated for 24 hr, serum starved for 16 hr, and treated with EGF (20 ng/ml) for 1 hr, related to figure 4f. (**g**) Relative mRNA levels of *CTGF* and *CYR61* in EGFR-overexpressing HEK293A cells transfected with MOB1 WT, 3YF, and 8YF. Data is same with figure 4f and S4f, but comparing only EGF-treated cells. (**h**) Immunoblot of HA, pEGFR (Y1068), EGFR, pLATS1 (T1079), LATS1, β-actin. EGFR-overexpressing HEK293A

cells were transfected with vector, HA-MOB1 WT or 8YF plasmid and incubated for 24 hr, serum starved for 16 hr, and treated with EGF (20 ng/ml) for 1 hr, related to figure 4f. Student's t-test and ANOVA with Tukey-Kramer post hoc test were used. Mean±SEM (**c**, **f**, **g**); ***, $P < 0.001$; **, $P < 0.01$; *, $P < 0.05$.

a

Rank	Oncogenic signatures gene set	NES	NOM p-val	FDR q-val
1	RPS14_DN.V1_DN	-3.05	0.000	0.000
2	CSR_LATE_UP.V1_UP	-2.93	0.000	0.000
3	RB_P107_DN.V1_UP	-2.84	0.000	0.000
4	VEGF_A_UP.V1_DN	-2.81	0.000	0.000
5	HOXA9_DN.V1_DN	-2.71	0.000	0.000
6	PRC2_EZH2_UP.V1_DN	-2.62	0.000	0.000
7	PRC2_EED_UP.V1_DN	-2.57	0.000	0.000
8	E2F1_UP.V1_UP	-2.49	0.000	0.000
9	GCNP_SHH_UP_LATE.V1_UP	-2.31	0.000	0.000
10	DUPONT: YAP	-2.25	0.000	0.000
11	CORDENONSI_YAP_CONSERVED_SIGNATURE	-2.10	0.000	0.001
12	SRC_UP.V1_DN	-2.05	0.000	0.002
13	MTOR_UP.V1_UP	-1.89	0.004	0.010
14	GCNP_SHH_UP_EARLY.V1_UP	-1.85	0.004	0.013
15	RB_P130_DN.V1_UP	-1.84	0.000	0.015
16	KRAS.300_UP.V1_UP	-1.83	0.005	0.014
17	CSR_EARLY_UP.V1_UP	-1.83	0.000	0.014
18	MYC_UP.V1_UP	-1.72	0.016	0.032
19	ESC_J1_UP_LATE.V1_DN	-1.69	0.009	0.037
20	KRAS.600_UP.V1_UP	-1.69	0.010	0.035

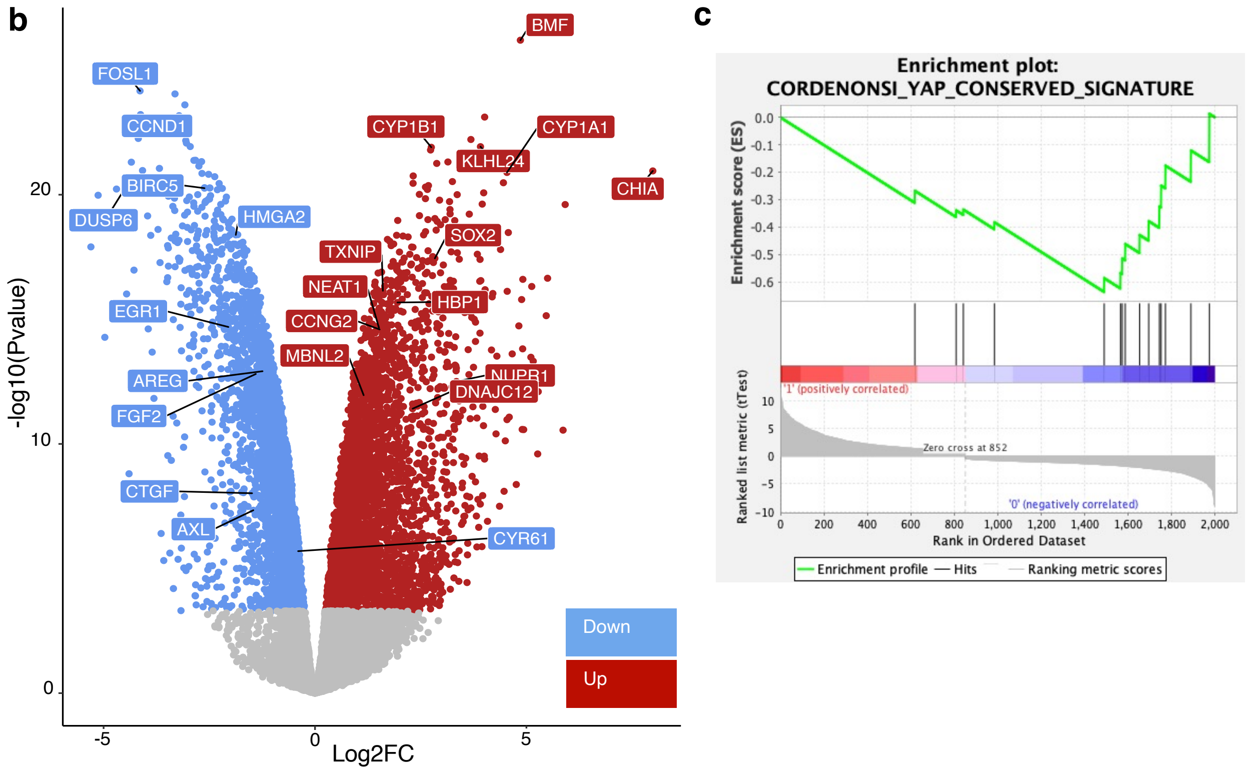

Supplementary Figure S5

(**a**) The top 20 enriched oncogenic signatures gene sets from RNA-seq data analysis of HCC827 cells. Cells were treated with vehicle or erlotinib (1 μM) for 24 hr. YAP-regulated signatures gene sets are highlighted in red, related to figure 5c. (**b**) Volcano plot showing that representative YAP/TAZ-regulated genes are reduced (shown as "down"). The genes highlighted in red are consistent with the ones previously reported as upregulated by erlotinib treatment[31], related to figure 5d. (**c**) Enrichment plots of YAP conserved signatures, related to figure 5e.

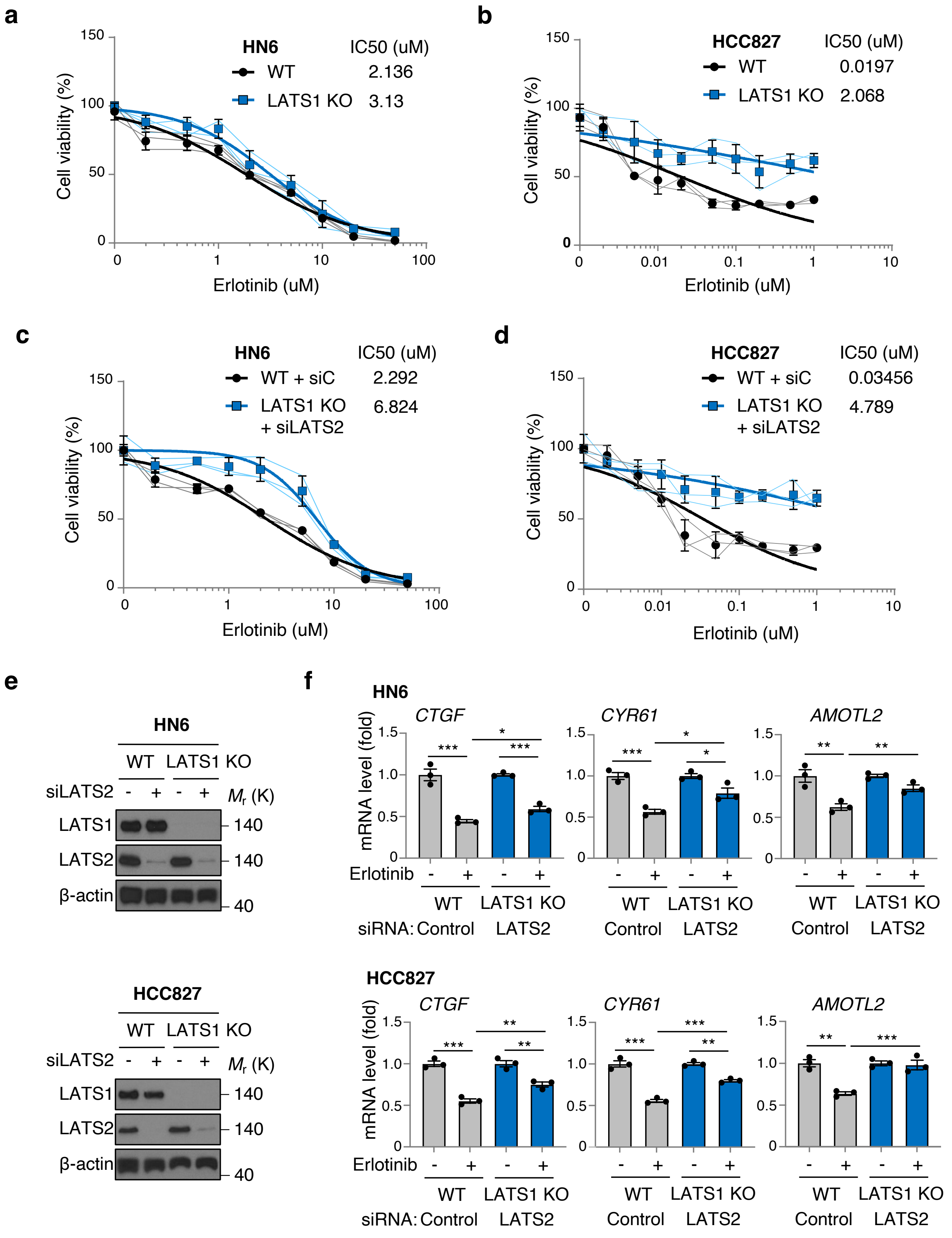
a
HN6
IC50 (uM)
WT 2.136
LATS1 KO 3.13
Cell viability (%)
Erlotinib (uM)
b
HCC827
IC50 (uM)
WT 0.0197
LATS1 KO 2.068
Cell viability (%)
Erlotinib (uM)
c
HN6
IC50 (uM)
WT + siC 2.292
LATS1 KO + siLATS2 6.824
Cell viability (%)
Erlotinib (uM)
d
HCC827
IC50 (uM)
WT + siC 0.03456
LATS1 KO + siLATS2 4.789
Cell viability (%)
Erlotinib (uM)
e
HN6
WT LATS1 KO
siLATS2
Mr (K)
LATS1
LATS2
β-actin
140
140
40
HCC827
WT LATS1 KO
siLATS2
Mr (K)
LATS1
LATS2
β-actin
140
140
40
f
HN6
CTGF
CYR61
AMOTL2
mRNA level (fold)
Erlotinib
WT LATS1 KO
siRNA: Control LATS2
HCC827
CTGF
CYR61
AMOTL2
mRNA level (fold)
Erlotinib
WT LATS1 KO
siRNA: Control LATS2

Supplementary Figure S6

(**a**, **b**) Cell viability of WT or LATS1 KO WSU-HN6 and HCC827 cells. Cells were treated with erlotinib as indicated concentration for 3 days. (**c**, **d**) Cell viability of WT or LATS1 KO HN6 and HCC827 cells, transfected with siLATS2. (**e**) Immunoblot of LATS1, LATS2, β-actin. (**f**) Relative mRNA levels of *CTGF*, *CYR61* and *AMOTL2*. Cells were transfected with siRNA for control or LATS2 and incubated for 48 h, then treated with Erlotinib (1 μM) for 2 hr, related to figure 6a-c. ANOVA with Tukey-Kramer post hoc test was used. Mean±SEM (**f**); ***, $P < 0.001$; **, $P < 0.01$; *, $P < 0.05$.

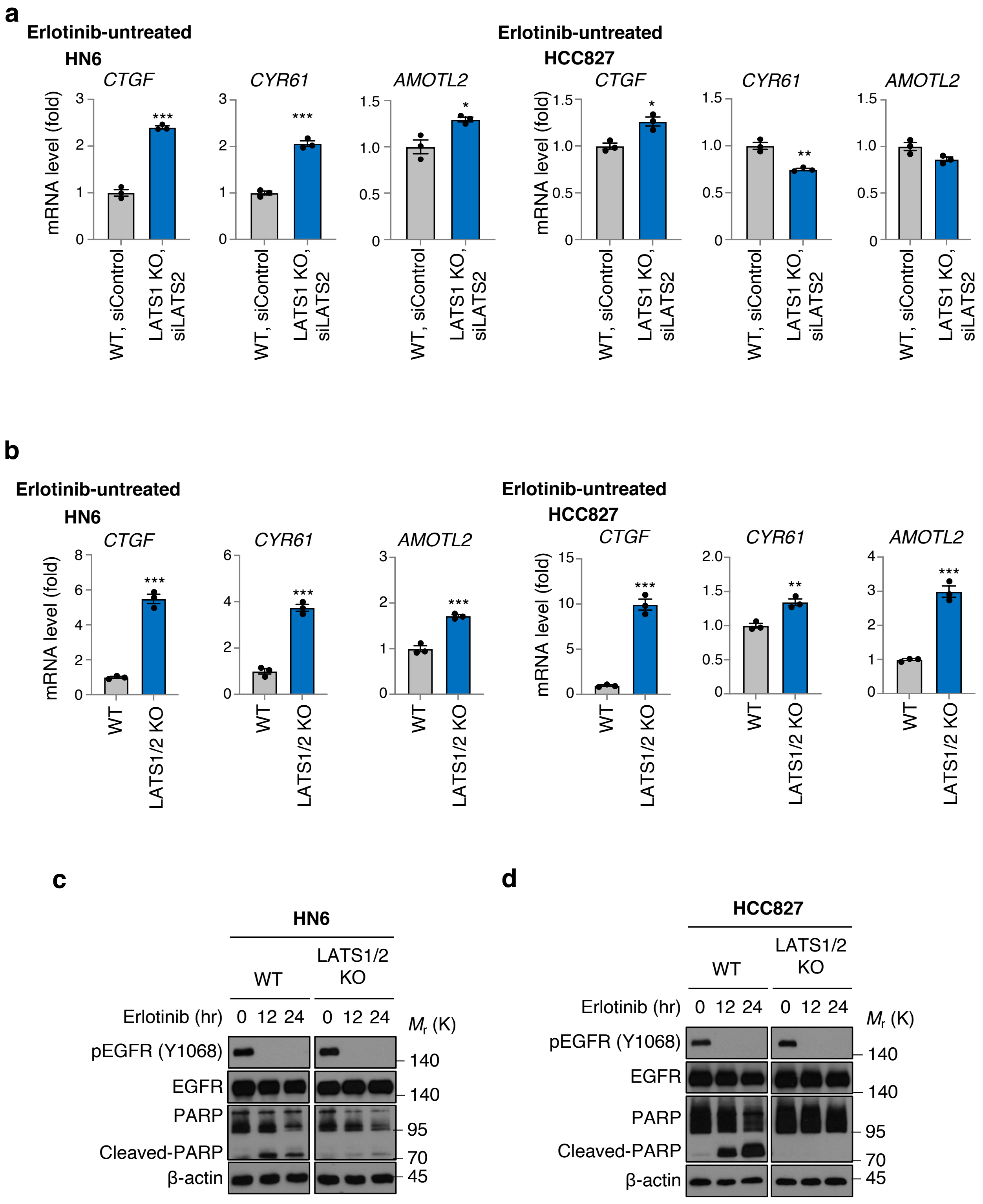
a
Erlotinib-untreated
HN6
CTGF
CYR61
AMOTL2
mRNA level (fold)
WT, siControl
LATS1 KO, siLATS2
Erlotinib-untreated
HCC827
b
Erlotinib-untreated
HN6
WT
LATS1/2 KO
Erlotinib-untreated
HCC827
c
HN6
WT
LATS1/2 KO
Erlotinib (hr)
0 12 24 0 12 24
M_r (K)
pEGFR (Y1068)
EGFR
PARP
Cleaved-PARP
β-actin
140
140
95
70
45
d
HCC827

Supplementary Figure S7

(**a**) Relative mRNA levels of *CTGF*/*CYR61*/*AMOTL2* in WT and LATS1 KO HN6 and HCC827 cells, transfected with siLATS2. Data is same with supplementary figure S6f, but only erlotinib-untreated cells are compared. (**b**) Relative mRNA levels of *CTGF*/*CYR61*/*AMOTL2* in WT and LATS1/2 KO HN6 and HCC827 cells. Data is same with figure 6b, but only erlotinib-untreated cells are compared. (**c**, **d**) Immunoblot of pEGFR (Y1068), EGFR, PARP (also detecting cleaved-PARP), β-actin. Cells were treated with vehicle or erlotinib (1 μM) for 0, 12, 24 hr. Student's t-test was used. Mean ± SEM (**a**, **b**); ***, $P < 0.001$; **, $P < 0.01$; *, $P < 0.05$.

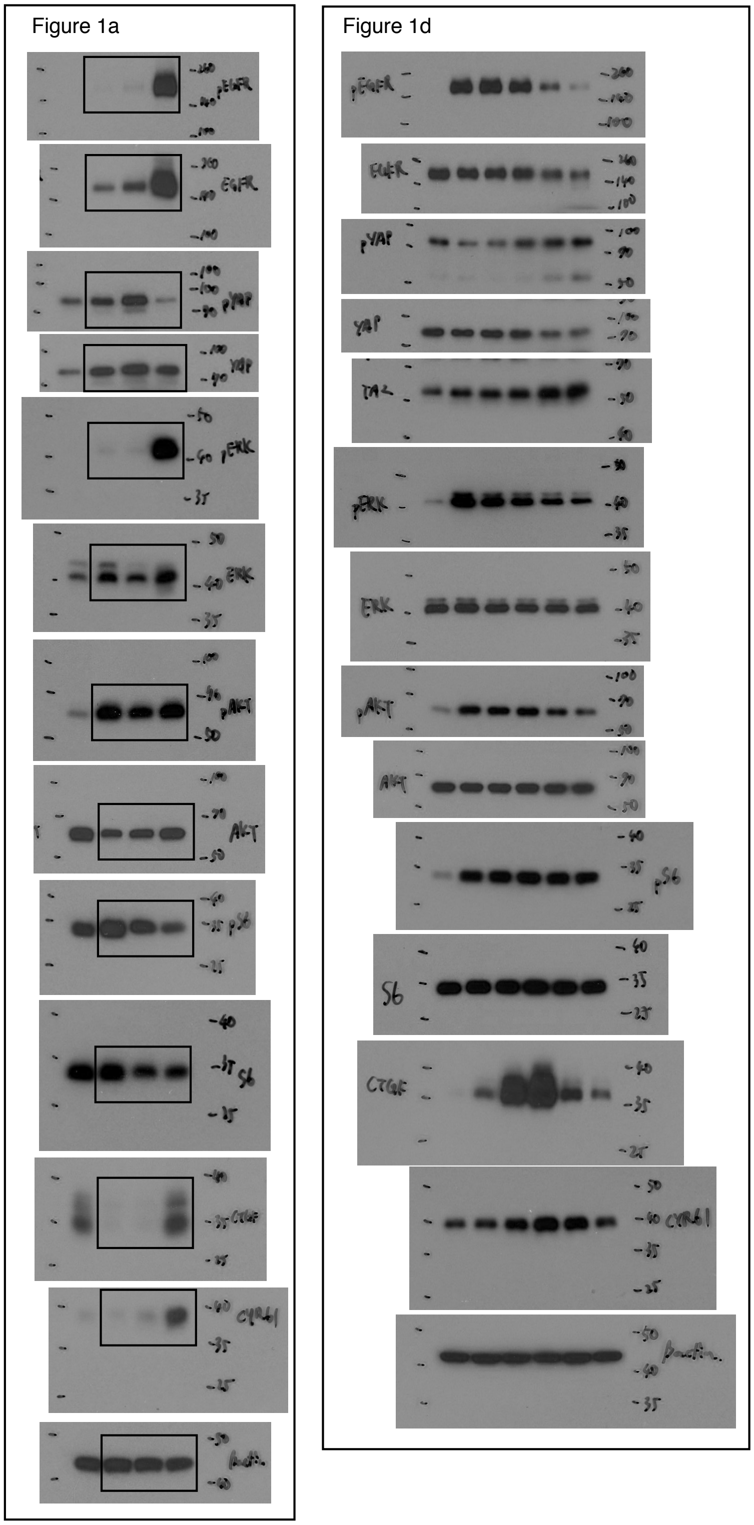

Supplementary Figure S8

Uncropped blot corresponding to Figs. 1a and d.

Figure 1f

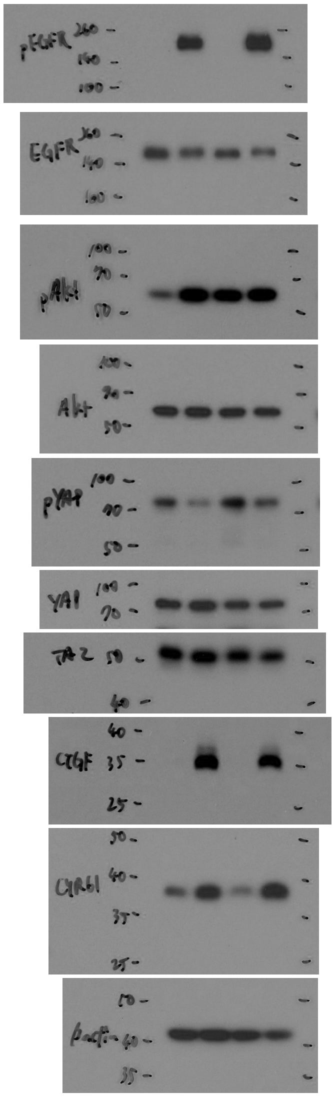

Figure 1g

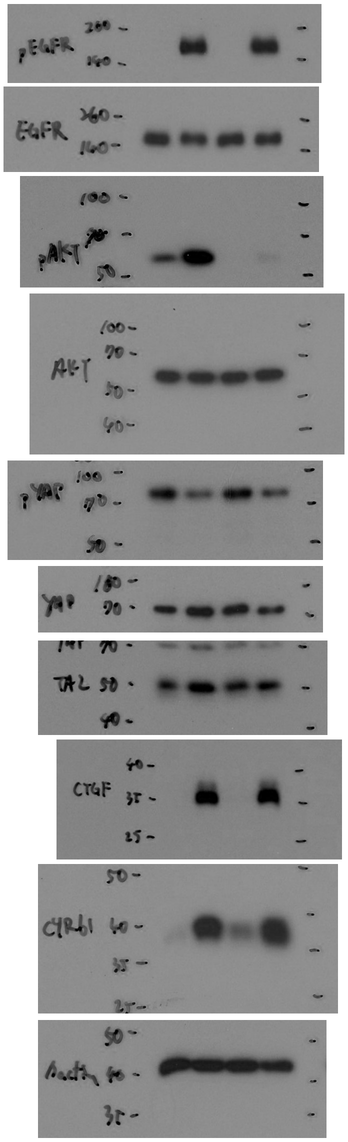

Supplementary Figure S9

Uncropped blot corresponding to Figs. 1f and g.

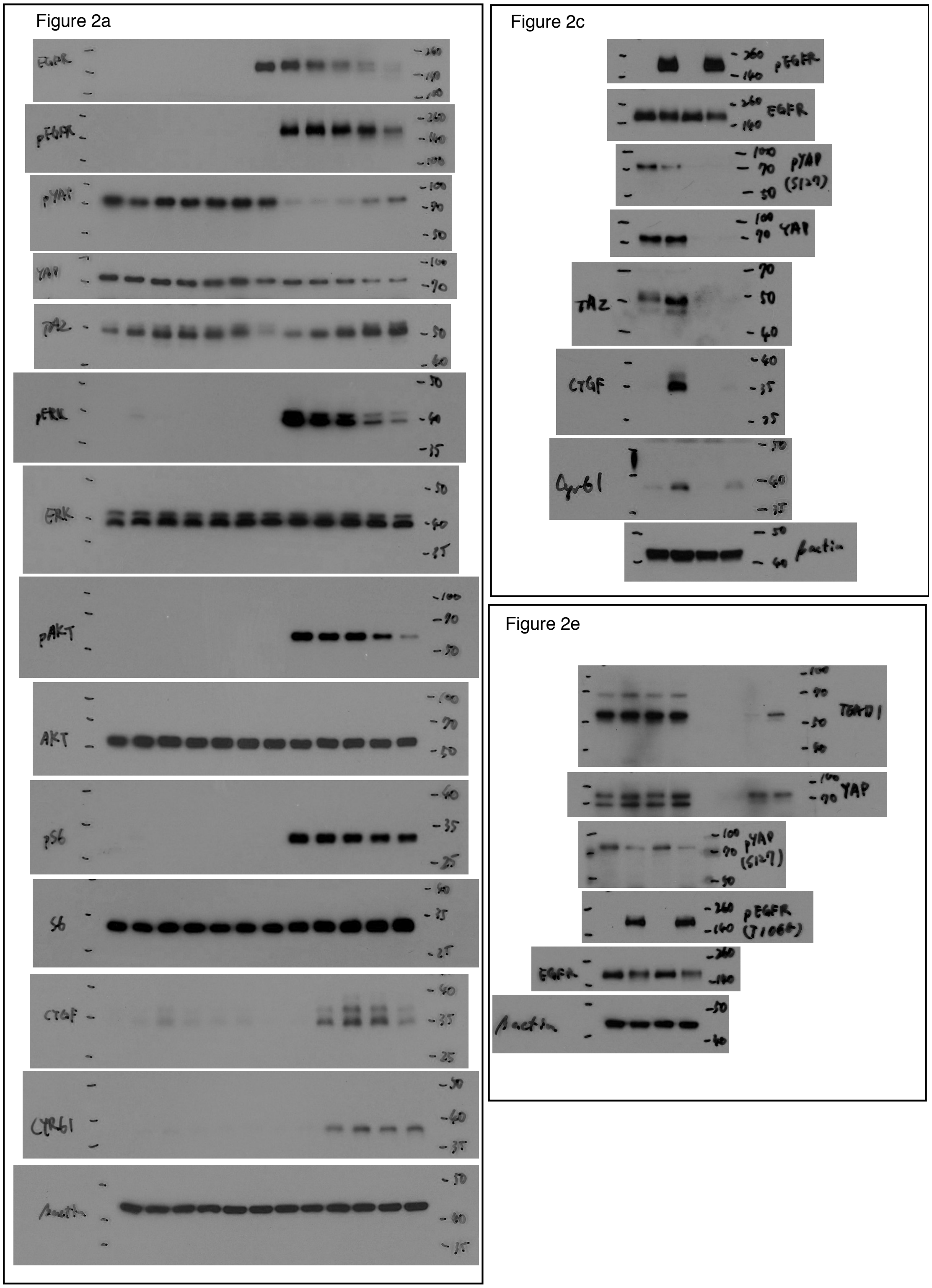

Supplementary Figure S10

Uncropped blot corresponding to Figs. 2a, c and e.

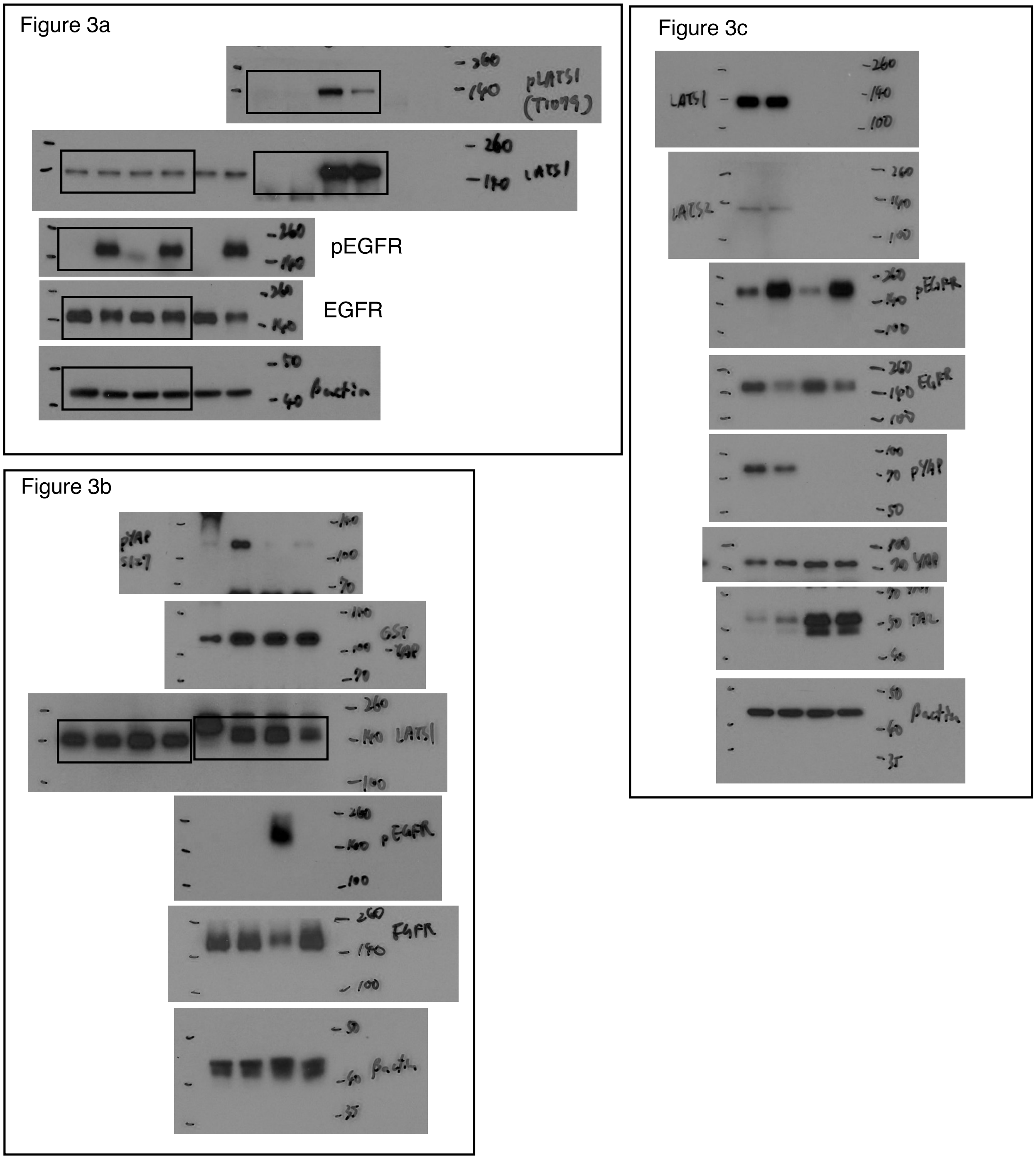

Supplementary Figure S11

Uncropped blot corresponding to Figs. 3a-c.

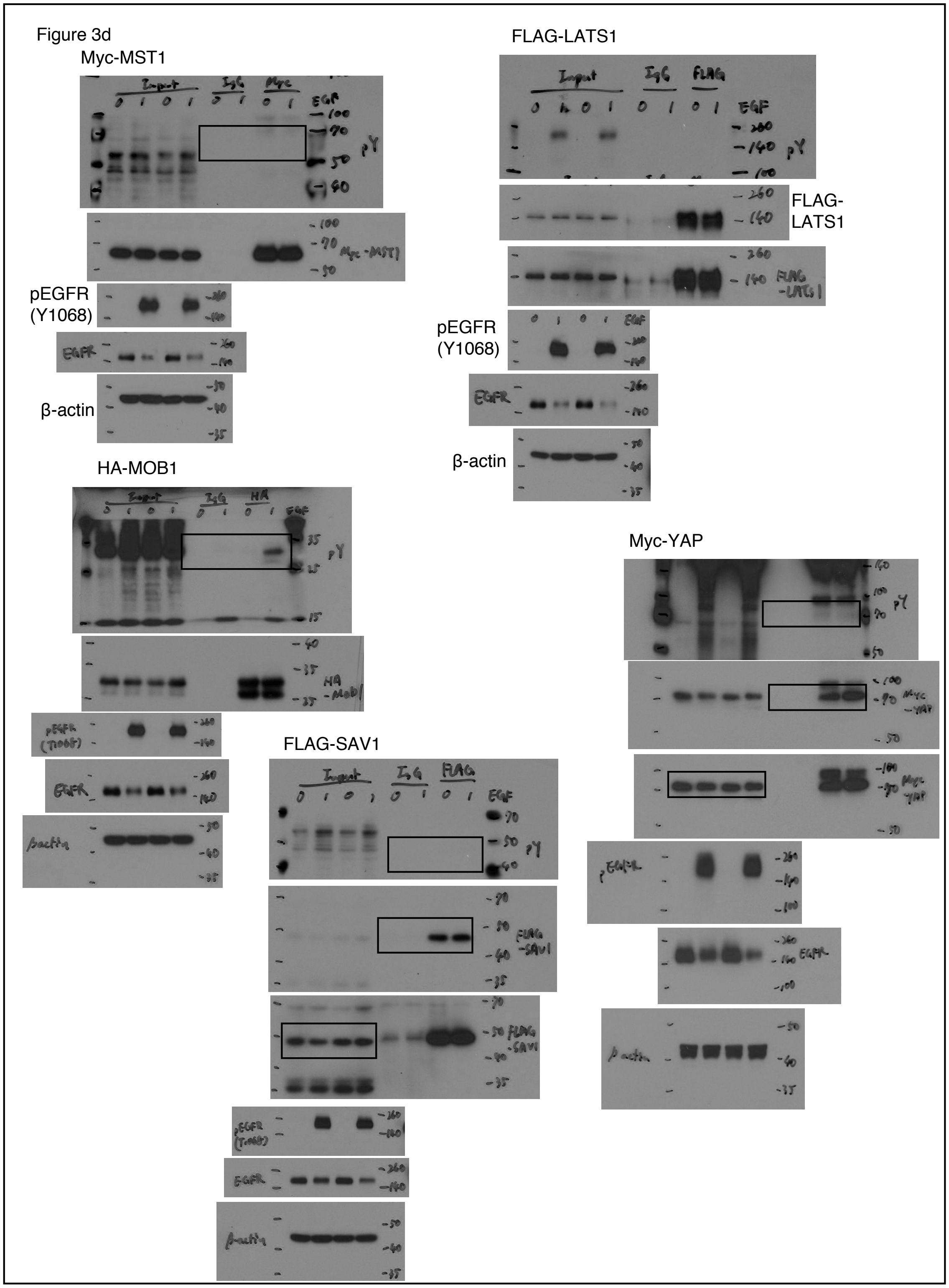

Supplementary Figure S12

Uncropped blot corresponding to Fig. 3d.

Figure 3e

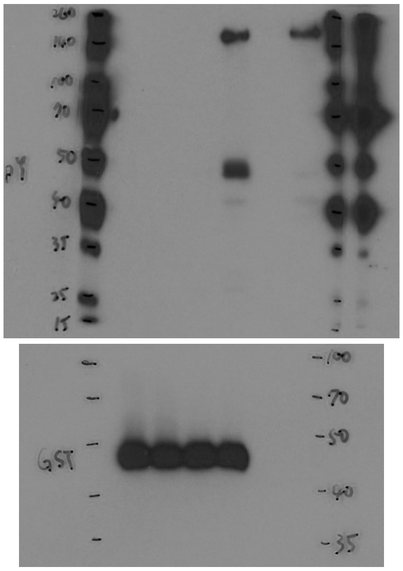

Figure 3f

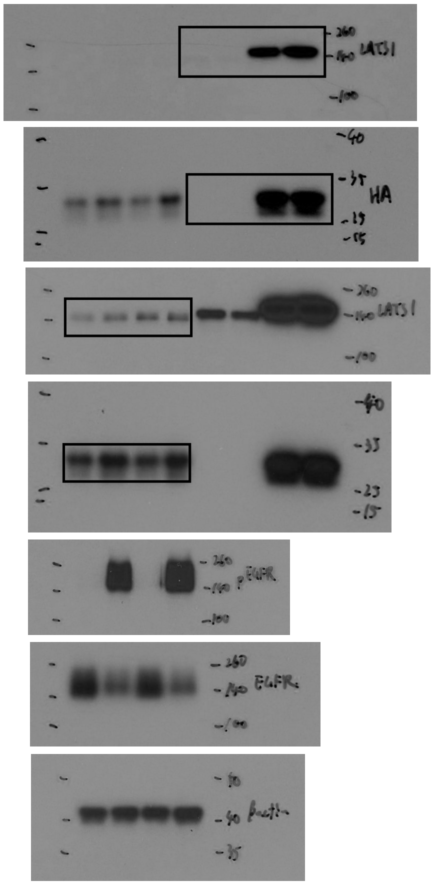

Figure 3g

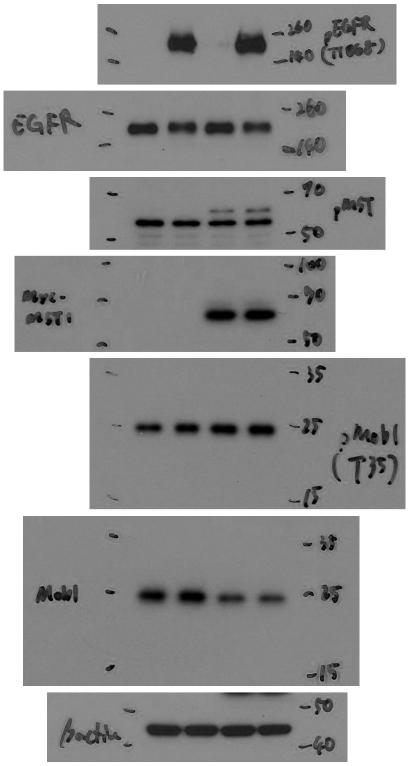

Supplementary Figure S13

Uncropped blot corresponding to Figs. 3e-g.

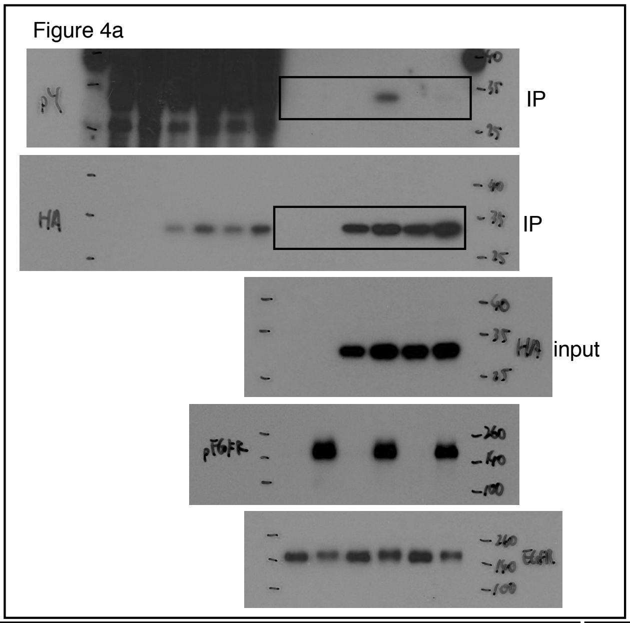

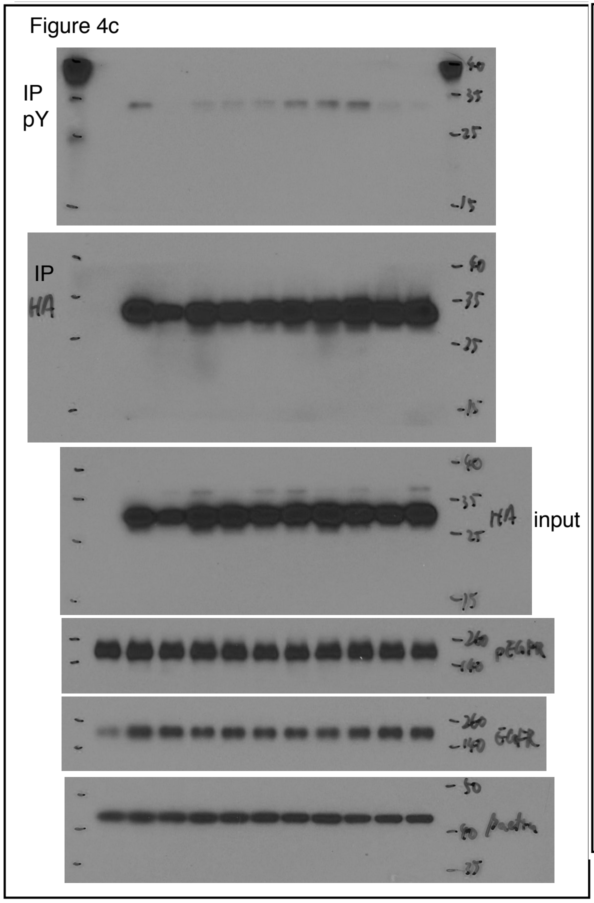

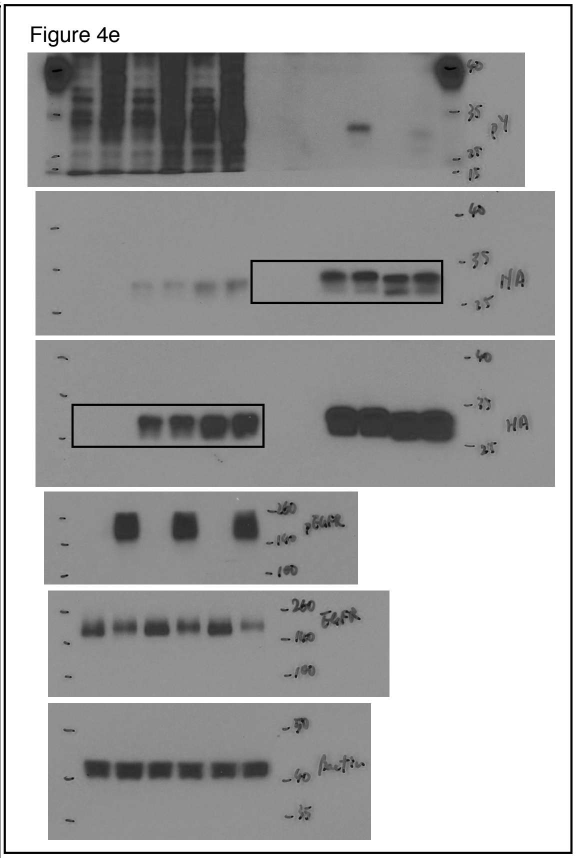

Supplementary Figure S14

Uncropped blot corresponding to Figs. 4a, c and e.

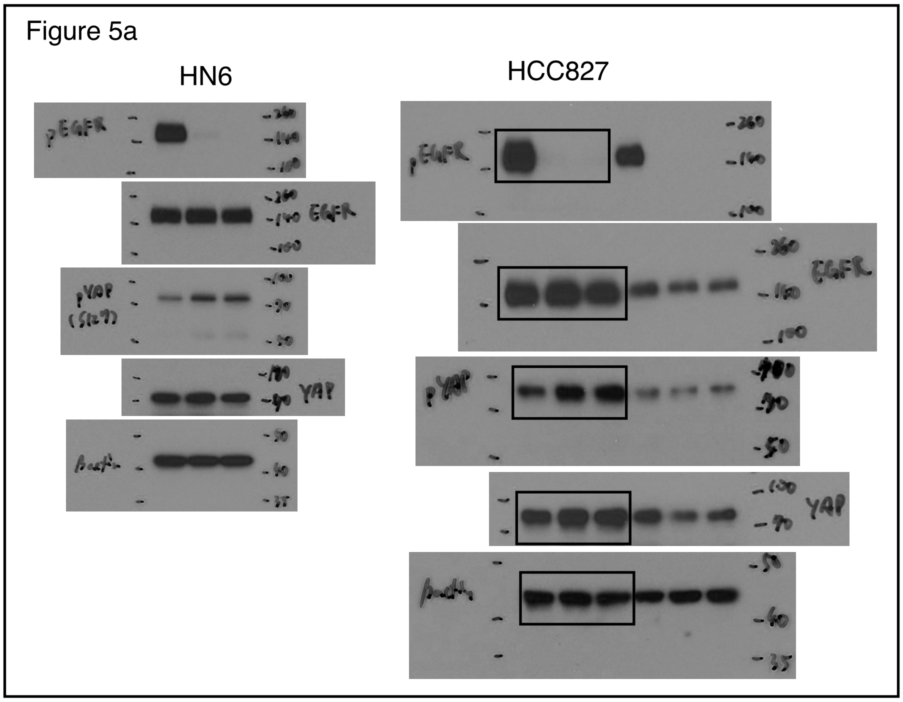

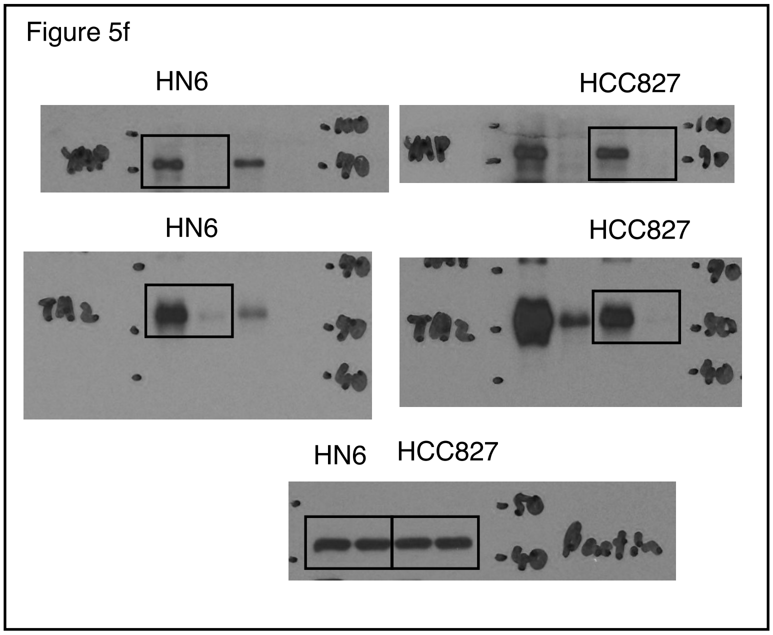

Supplementary Figure S15

Uncropped blot corresponding to Figs. 5a and f.

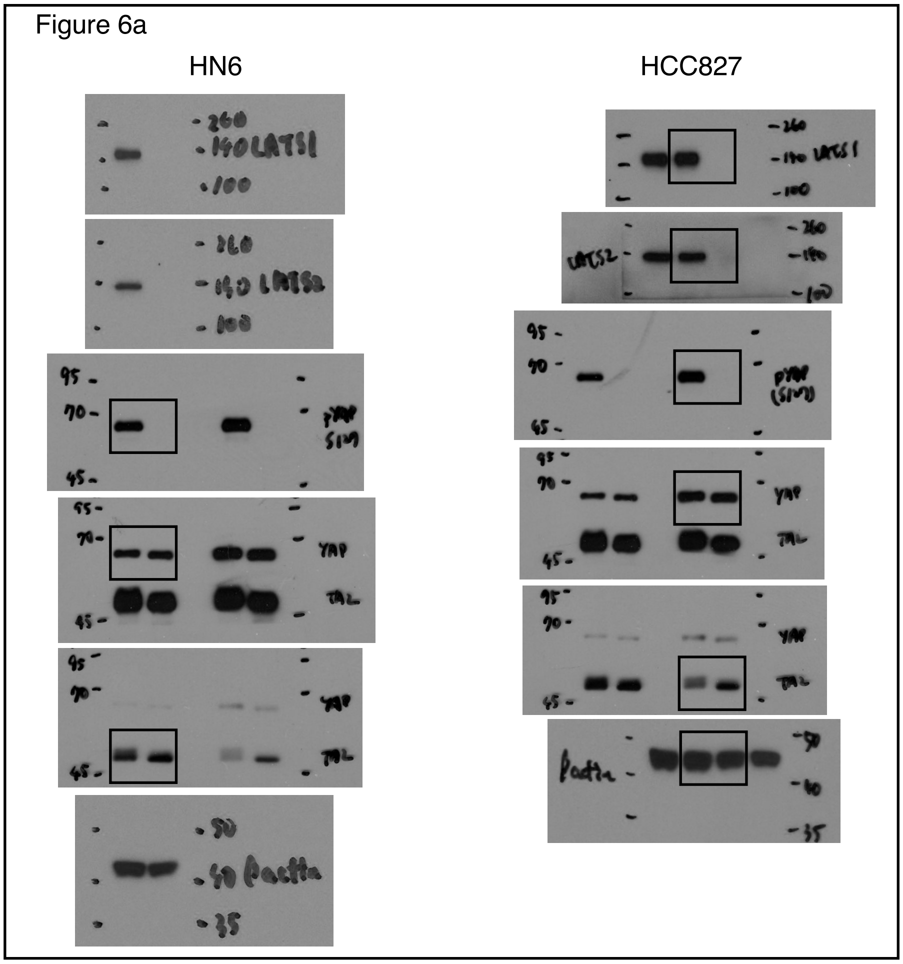

Supplementary Figure S16

Uncropped blot corresponding to Fig. 6a.

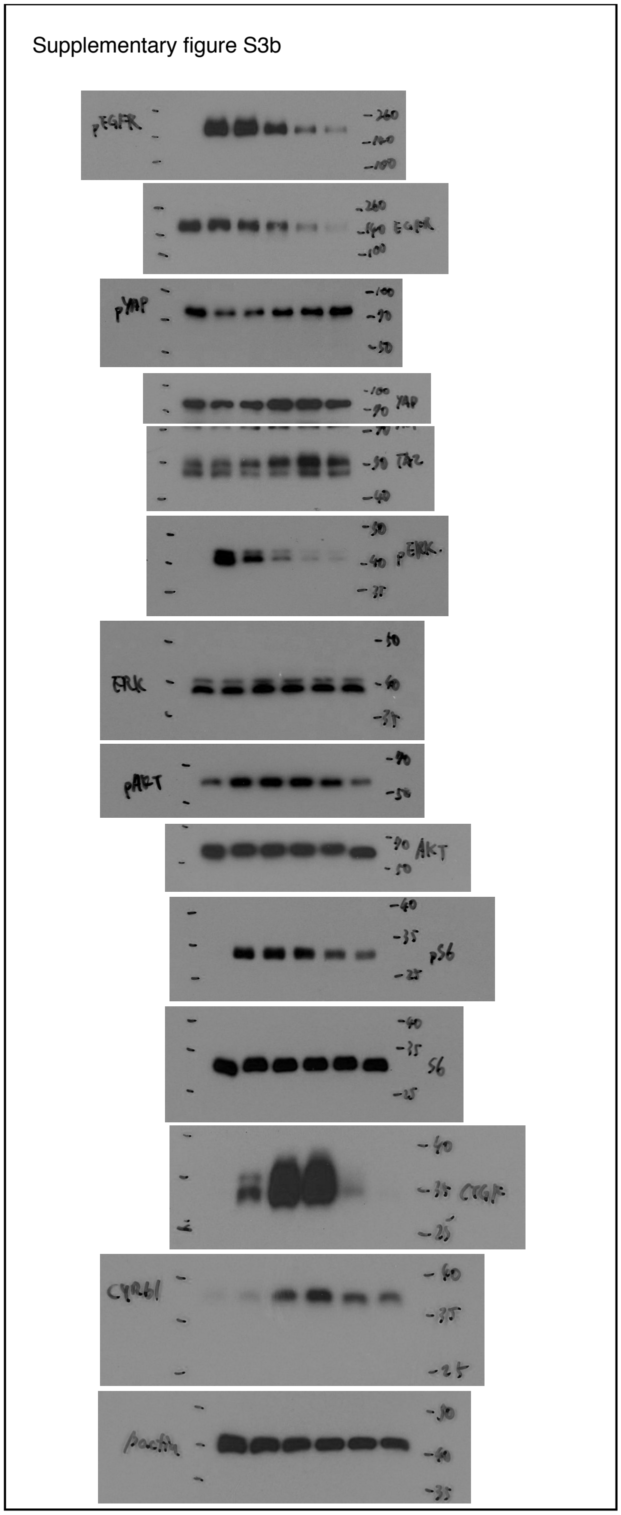

Supplementary Figure S17

Uncropped blot corresponding to Supplementary Fig. S3b.

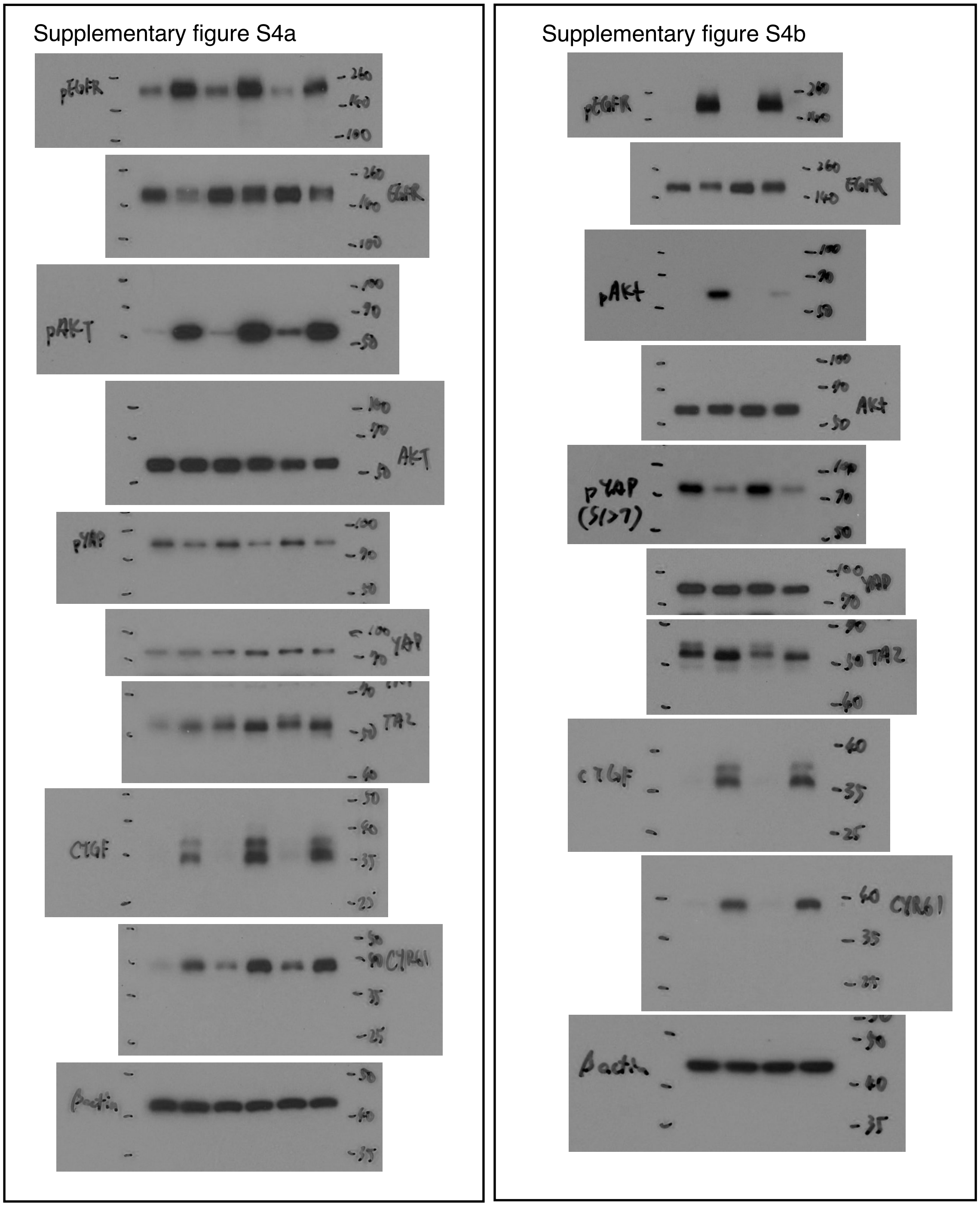

Supplementary Figure S18

Uncropped blot corresponding to Supplementary Figs. S4a and b.

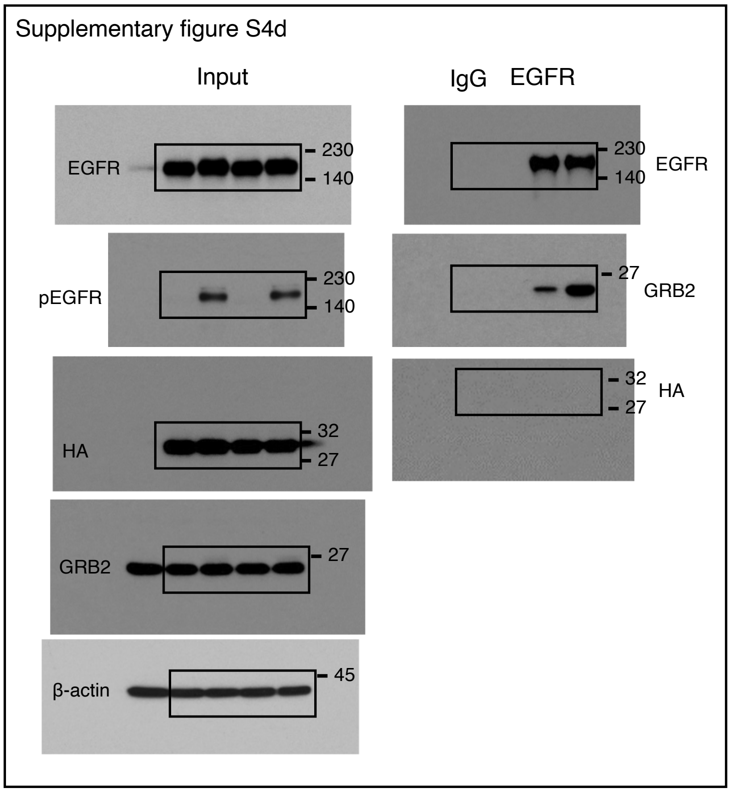

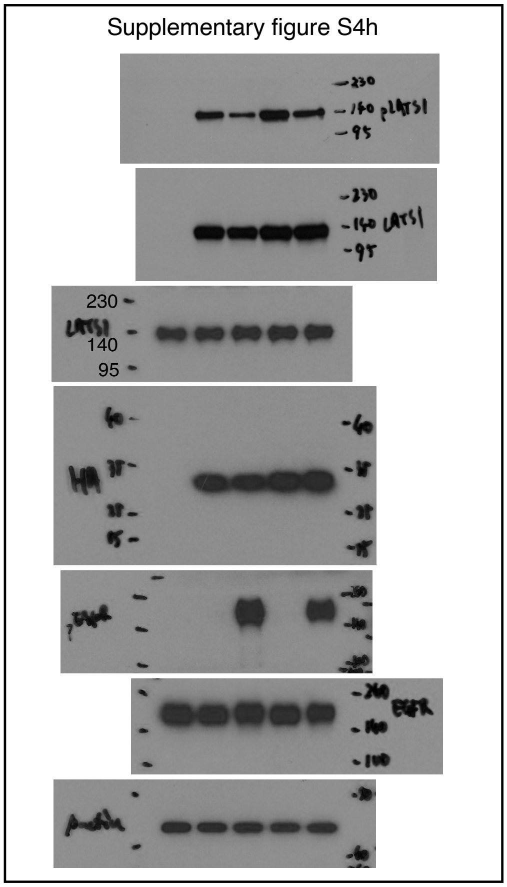

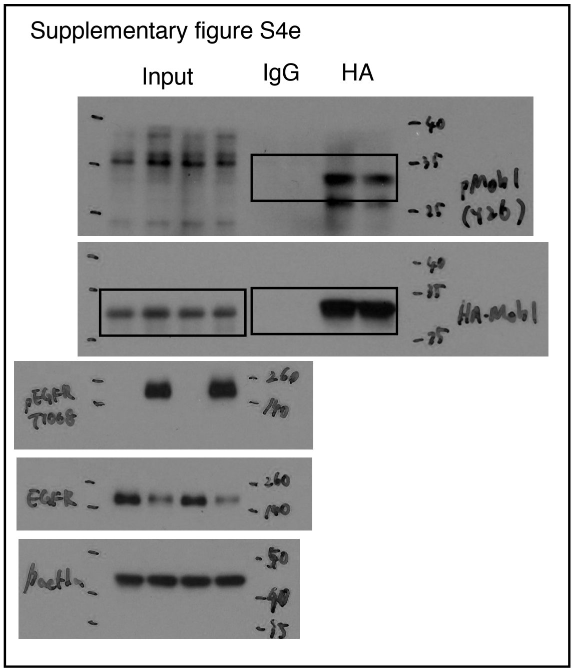

Supplementary Figure S19

Uncropped blot corresponding to Supplementary Figs. S4d, e and h.

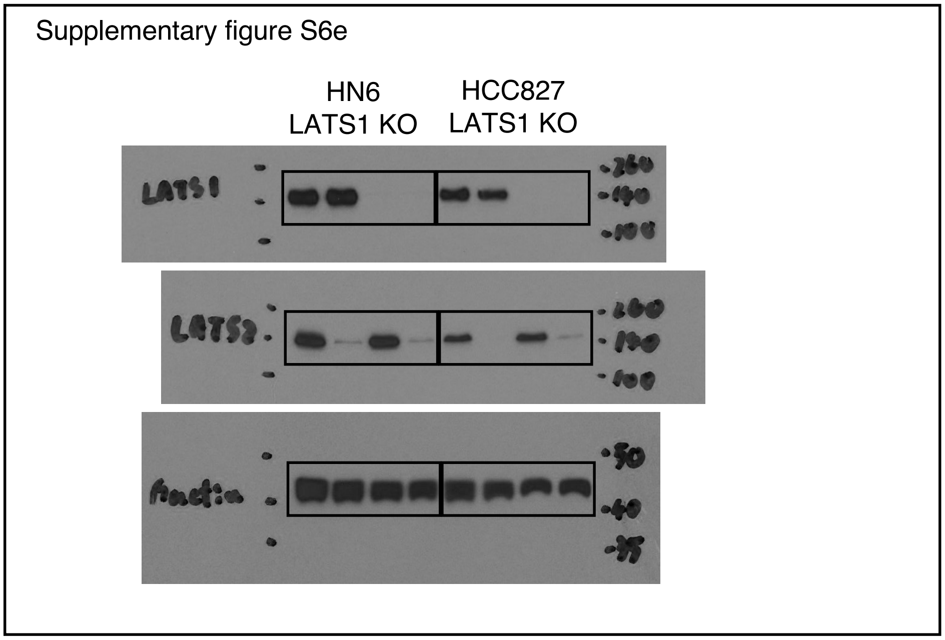

Supplementary Figure S20

Uncropped blot corresponding to Supplementary Fig. S6e.

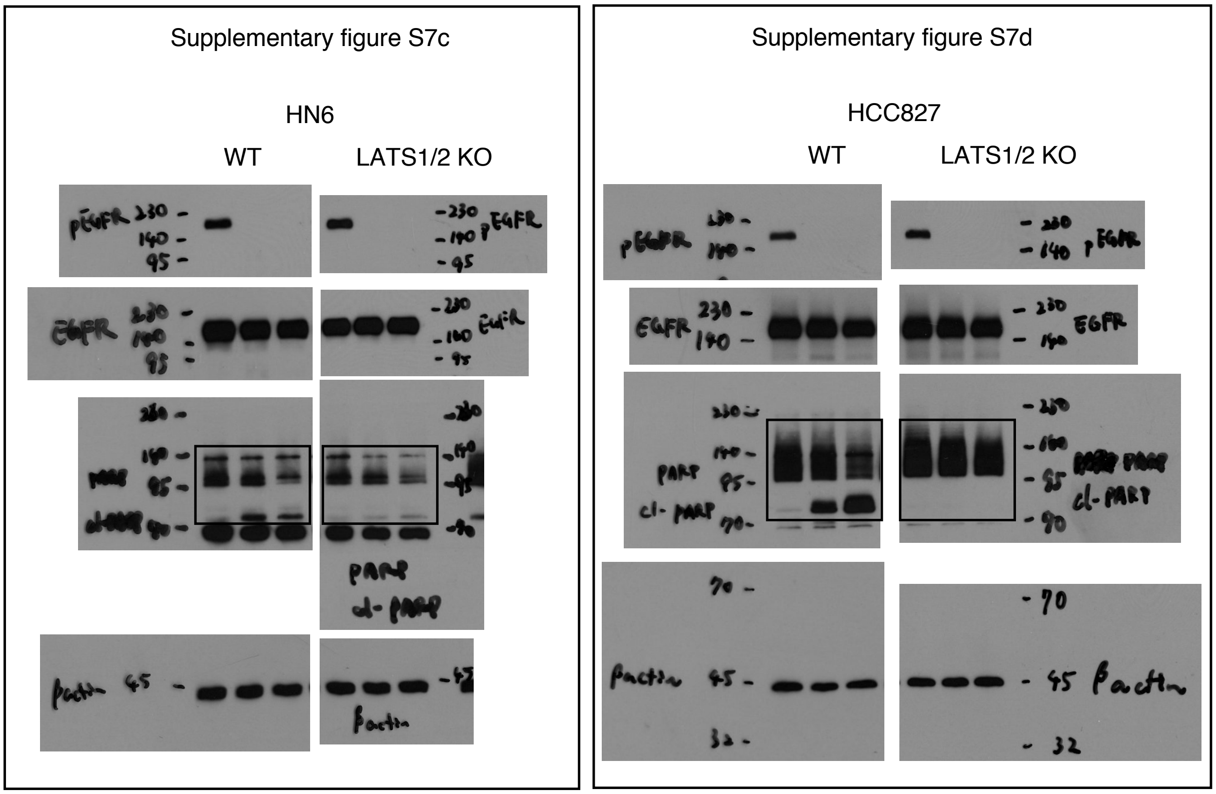

Supplementary Figure S21

Uncropped blot corresponding to Supplementary Figs. S7c and d.
