# Supplementary material for: EGFR Regulates the Hippo pathway by promoting the tyrosine phosphorylation of MOB1
Source: Commun Biol. 2021 Nov 1;4:1237. doi: 10.1038/s42003-021-02744-4 (PMC8560880; doi:10.1038/s42003-021-02744-4)
Supplement: Supplementary file 2 — Description of Additional Supplementary Files [file 42003_2021_2744_MOESM2_ESM.pdf]

Description of Additional Supplementary Files

File name: Supplementary Data 1.
Description: Source data underlying Figs 1b, e, 2b, d, 4f, 5b, g, 6b, c, 7e and Supplementary Figs S3c, S4c, f, g, S6a-d, f, S7a, b.
